# One-time sintering process to synthesize ZrO_2_-coated LiMn_2_O_4_ materials for lithium-ion batteries†

**DOI:** 10.1039/c8ra01421c

**Published:** 2018-05-08

**Authors:** Gang Li, Xu Chen, Yafei Liu, Yanbin Chen, Wensheng Yang

**Affiliations:** State Key Laboratory of Chemical Resource Engineering, Beijing University of Chemical Technology Beijing 100029 P. R. China yangws@mail.buct.edu.cn; Beijing Easpring Material Technology Co., Ltd. Beijing 100160 P. R. China

## Abstract

Herein, different amounts of ZrO_2_-coated LiMn_2_O_4_ materials are successfully prepared by one-time sintering ZrO_2_-coated Mn_3_O_4_ and Li_2_CO_3_. Scanning and transmission electron microscopy results confirm that the ZrO_2_ coating layer on the surface of Mn_3_O_4_ can still be maintained on the surface of the final LiMn_2_O_4_ particles even after long-term high-temperature heat-treatment. Three key factors to realize ZrO_2_-coated LiMn_2_O_4_ materials *via* the one-time sintering process are as follows: (i) the Mn_3_O_4_ precursor is coated by ZrO_2_ in advance; (ii) the ionic radius of Zr^4+^ is much larger than those of Mn^3+^ and Mn^4+^; (iii) the pre-calcination temperature is set in the reaction temperature range between Li_2_CO_3_ and Mn_3_O_4_ and lower than that between Li_2_CO_3_ and ZrO_2_. The 3 wt% ZrO_2_-coated LiMn_2_O_4_ material exhibits excellent electrochemical properties with an initial specific discharge capacity of 118.8 mA h g^−1^ and the capacity retention of 90.1% after 400 cycles at 25 °C and 88.9% after 150 cycles at 55 °C. Compared with the conventional coating method, the one-time sintering process to synthesize ZrO_2_-coated LiMn_2_O_4_ materials is very simple, low-cost, environmentally friendly, and easy to scale up for large-scale industrial production, which also provides a valuable reference for preparing other coating-type cathode materials for lithium-ion batteries.

## Abstract 2

A facile one-time sintering process was used to synthesize ZrO_2_-coated LiMn_2_O_4_ to greatly enhance its cycling performance.

## Graphical abstract

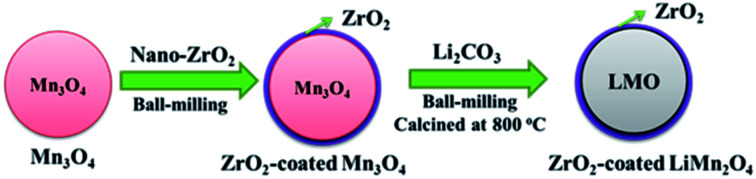

## Introduction

Rechargeable lithium-ion batteries (LIBs) have achieved great success as the power sources for portable electronic devices due to their high energy density and long cycle life. Recently, the demand for LIBs has been increasingly shifting from small portable power systems to electric vehicles (EVs) and large-scale energy storage systems (ESSs).^1,2^ The safety and cost issues are mostly concerned for EVs and ESSs.^3,4^ LiMn_2_O_4_ is one of the most suitable cathode materials owing to its high abundance, low material cost, and high safety.^5^ However, the practical application of LiMn_2_O_4_ is greatly limited due to their poor cycling performance, especially at elevated temperatures (≥55 °C).^6^ The reasons can be summarized as follows: (1) the dissolution of Mn^2+^ from cathode to electrolyte *via* a disproportionation reaction (2Mn^3+^_solid_ → Mn^4+^_solid_ + Mn^2+^_electrolyte_);^7–9^ (2) structural transformation from cubic to tetragonal phase, induced by the Jahn–Teller distortion of Mn^3+^;^10,11^ (3) the electrolyte decomposition on the electrode surface at high voltage.^12^

To solve the above problems, two strategies, namely the doping and coating techniques, are usually employed to improve the room-temperature (RT) and high-temperature (HT) cycling performance of LiMn_2_O_4_ materials. Partial substitution of Mn^3+^ with other metal ions, such as Li^+^, Al^3+^, Co^3+^, and Cr^3+^, can stabilize the structure of LiMn_2_O_4_ materials, which is attributed to the strong metal–oxygen bonds formed by the doping metals.^5,13–15^ With regard to the synthetic operations, the doping elements can be easily added in the precursor-synthesizing or the raw-material-mixing processes.^16–20^ Therefore, no extra processes are needed and the production cost would not be greatly increased.

The coating technology is another effective strategy to improve the cycling performance of LiMn_2_O_4_ materials. The coating layer can protect the inner active materials from attack by the acidic species such as HF and oxidation of carbonate solvents at high voltages.^21^ Many electrochemical inert materials^12,22–24^ such as ZrO_2_, Al_2_O_3_, TiO_2_, AlPO_4_, AlF_3_ and many coating methods^6,25–29^ such as sol–gel, wet-coating, spray drying, atomic layer deposition (ALD) have been widely studied. Until now, the coating-type LiMn_2_O_4_ materials are usually synthesized by sintering two times: the pristine LiMn_2_O_4_ is firstly prepared by calcination at ∼800 °C, and then the LiMn_2_O_4_ is coated with some inert materials and followed by re-calcination between 400 °C and 600 °C. Although the coating techniques can effectively improve the cycling performance, they still have some drawbacks. For example, the production cost of ALD is very high, the sol–gel and spray drying methods are of high-energy consumption, and wet-coating method would generate a large amount of wastewater. Therefore, the industrial applications of the current coating techniques are greatly hindered by the complicated or high-cost coating processes.^30,31^ Consequently, a simple, low-cost, environmental friendly, and easily scaled-up coating technique is urgent to be developed to solve the above problems.

In this work, a one-time sintering process was employed to synthesize ZrO_2_-coated LiMn_2_O_4_ materials. Scanning and high resolution transmission electron microscope (SEM and HRTEM) results clearly demonstrate that the LiMn_2_O_4_ particles can be successfully coated by ZrO_2_*via* the one-time sintering process. The electrochemical measurements demonstrate that the RT and HT cycling performances of ZrO_2_-coated LiMn_2_O_4_ materials are greatly enhanced. This one-time sintering process to synthesize ZrO_2_-coated LiMn_2_O_4_ materials is very simple, low-cost, and environmental friendly, which can promote its practical application. Besides, we systematically investigated the key influence factors in the synthesis of ZrO_2_-coated LiMn_2_O_4_ materials *via* the one-time sintering process, which can provide a valuable reference for synthesizing other coating-type cathode materials for LIBs.

## Experimental

### Material preparation

The pristine and different amounts of ZrO_2_-coated LiMn_2_O_4_ materials were synthesized by a high-temperature solid-state reaction. The shape and particle size distribution of the raw materials of Mn_3_O_4_ (Nanjing Tianyuan Magnetic Material Co., Ltd.) and ZrO_2_ (Xuan Cheng Jing Rui New Material Co., Ltd.) can be seen in Fig. S1 and S2 in ESI.† The amount of ZrO_2_ was calculated based on the weight of the final LiMn_2_O_4_ material. Therefore, in order to synthesize 1, 3, and 5 wt% ZrO_2_-coated LiMn_2_O_4_, the adding amount of ZrO_2_ was 1.19, 3.56, and 5.93 wt% of the weight of Mn_3_O_4_, respectively.

The one-time sintering process to synthesize 3 wt% ZrO_2_-coated LiMn_2_O_4_ is as follows as an example: industrial grade Mn_3_O_4_ (100 g) and nano-sized ZrO_2_ (3.56 g) were firstly weighed and ball-milled for 1 h, and then battery-grade Li_2_CO_3_ (25.43 g, Shandong RuiFu Lithium Co., Ltd.) with the molar ratio of Li : Mn = 1.05 : 2 was added to the above mixed materials and further ball-milled for 3 h. Note that the mass ratio of the agate balls to the raw materials is 1 : 1, and the rotational speed of the planetary ball mill is 400 rpm. The obtained mixtures of different amounts of ZrO_2_-coated Mn_3_O_4_ & Li_2_CO_3_ was added into alumina crucible, then pre-heated at 550 °C for 5 h and calcined at 800 °C for 20 h in O_2_ atmosphere. Both of the heating and cooling rates are 3 °C min^−1^. The calcined samples were crushed down and sifted through a sieve of 325 meshes, thus the final products were obtained. The step for preparing ZrO_2_-coated Mn_3_O_4_ can be ignored in the synthesis of the pristine LiMn_2_O_4_.

In order to investigate the influence of the blending manners of raw materials on the uniformity of the coating layer on particle surface of the final materials, stoichiometric amount of Mn_3_O_4_, Li_2_CO_3_, and ZrO_2_ (Li : Mn = 1.05 : 2, molar ratio) are added and ball-milled at the same time. The detailed synthetic process can be seen in the ESI.† The obtained sample is denoted as LMO@ZrO_2_. Additionally, 3 wt% ZrO_2_-coated LiMn_2_O_4_ material was also synthesized by the conventional dry coating method to compare the coating effect of two coating methods.

Besides, SiO_2_ and TiO_2_ were also used as the coating materials to synthesize different amounts of SiO_2_- and TiO_2_-coated LiMn_2_O_4_ materials, respectively. The detailed synthetic process can be seen in the ESI.†

### Material characterization

The morphologies of the synthesized materials were observed using SEM (Supra 55, Zeiss, Germany) with an energy dispersive X-ray spectroscope (EDX, EDAX, Genesis 60, Germany). The thickness of ZrO_2_ coating layer was measured using Auger electron spectroscopy (AES, ULVAC-PHI, AES-PHI 700, Japan). Lattice structure and surface feature of samples were investigated using HRTEM (H-800, Hitachi, Japan). Powder X-ray diffraction measurements (XRD, D/MAX 2500, Rigaku, Japan) using Cu K_α_ radiation (*λ* = 0.154 nm) were employed to identify the crystalline phase of the synthesized materials. XRD data were obtained in the 2*θ* range of 10–80°, with a step size of 0.02°, and a count time of 4 s. From the XRD data, the lattice parameters were calculated by the least-squares method. The thermal and weight changes in the synthetic process were investigated with thermogravimetric and differential thermal analysis (TG-DTA, HCT-1, China). TG-DTA measurements of the mixed materials were performed between 25 °C and 850 °C at a heating rate of 3 °C min^−1^.

### Electrochemical measurement

The prepared powders were mixed with carbon black and polyvinylidene fluoride (PVDF) with a weight ratio of 80 : 10 : 10 in *N*-methyl-2-pyrrolidinone (NMP) to fabricate the positive electrodes. The obtained slurry was coated onto Al foil, followed by drying at 105 °C for 30 min, and roll-pressed in air. The electrodes were dried overnight at 100 °C in a vacuum oven prior to use. The areal mass loading of the active materials was ∼10 mg cm^−2^. The CR2032 coin cells were assembled in an argon-filled glove box with an electrolyte of 1 mol L^−1^ LiPF_6_ in EC-DMC-DEC (1 : 1 : 1 weight ratio) solution and a separator of Celgard 2400. The cells were aged for 6 h before the charge/discharge test performed on a LAND CT2001A test system (Wuhan LAND electronics Co. Ltd., China) in the voltage range of 3.0–4.3 V (*vs.* Li^+^/Li) at 25 °C and 55 °C. The first 4 cycles were performed at 0.2C (24 mA g^−1^), and then the following cycles were tested at 1C (120 mA g^−1^).

## Results and discussion

**Fig. 1:**
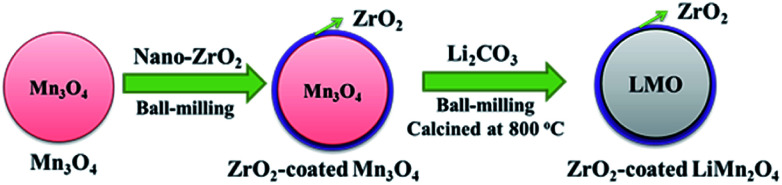
Schematic illustration for the one-time sintering process to synthesize ZrO_2_-coated LiMn_2_O_4_ materials.

As illustrated in Fig. 1, the one-time sintering process to synthesize ZrO_2_-coated LiMn_2_O_4_ materials includes two main procedures: the mixing and sintering processes. In the mixing process, Mn_3_O_4_ and nano-sized ZrO_2_ are firstly ball-milled for 1 h to prepare ZrO_2_-coated Mn_3_O_4_ materials, and then the stoichiometric amount of Li_2_CO_3_ is added to the above mixture and further ball-milled for 3 h. The purpose of the above process is to guarantee that most of ZrO_2_ particles are coated onto the surface of Mn_3_O_4_ and would not be coated onto the surface of Li_2_CO_3_ particles.

**Fig. 2:**
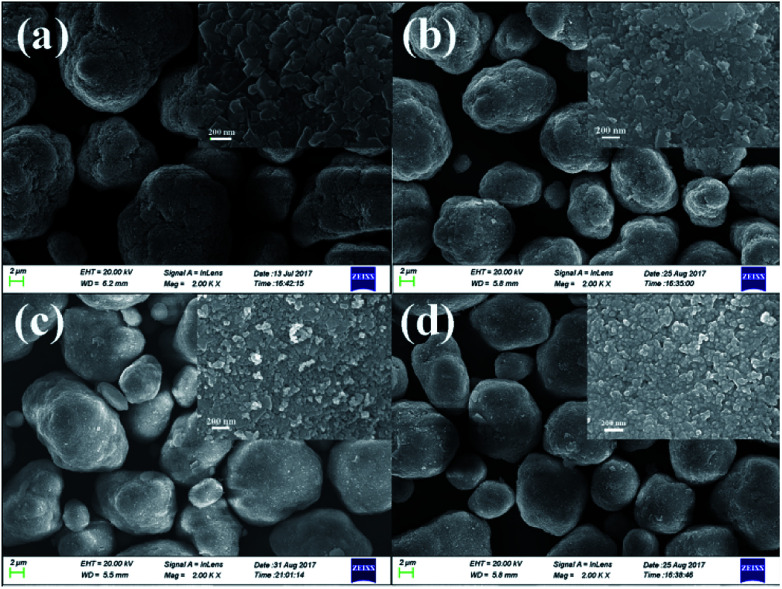
SEM images of (a) the pristine Mn_3_O_4_ and (b) 1.19 wt%, (c) 3.56 wt%, (d) 5.93 wt% ZrO_2_-coated Mn_3_O_4_ without any heat-treatment. The insets are the corresponding SEM images at high magnification.

Fig. 2 shows the SEM images of the pristine Mn_3_O_4_ and different amounts of ZrO_2_-coated Mn_3_O_4_ materials without any heat-treatment. As shown in Fig. 2a, the pristine Mn_3_O_4_ particles have spherical morphology and are composed of the octahedral primary particles with the particle size ranging from 100 to 200 nm. Additionally, we also notice that the Mn_3_O_4_ particles have a rough surface and there are many particle boundaries among the primary particles. Fig. 2b–d show the SEM images of different amounts of ZrO_2_-coated Mn_3_O_4_ materials. As illustrated from the low-magnification images in Fig. 2b–d, after ZrO_2_ coating, the particle boundary on the surface of Mn_3_O_4_ particles is gradually blurred with ZrO_2_ coating amount increasing. In addition, there are no nano-sized ZrO_2_ particles existing alone even when the coating amount is up to 5.93 wt%. The inset high-magnification SEM images shown in Fig. 2b–d clearly demonstrate that the surface of Mn_3_O_4_ is uniformly coated by many ZrO_2_ particles with the particle size of about 30 nm. The above results indicate that nano-sized ZrO_2_ can be easily coated on the surface of Mn_3_O_4_ particles through the simple dry coating method without adding any dispersant such as alcohol or water.

**Fig. 3:**
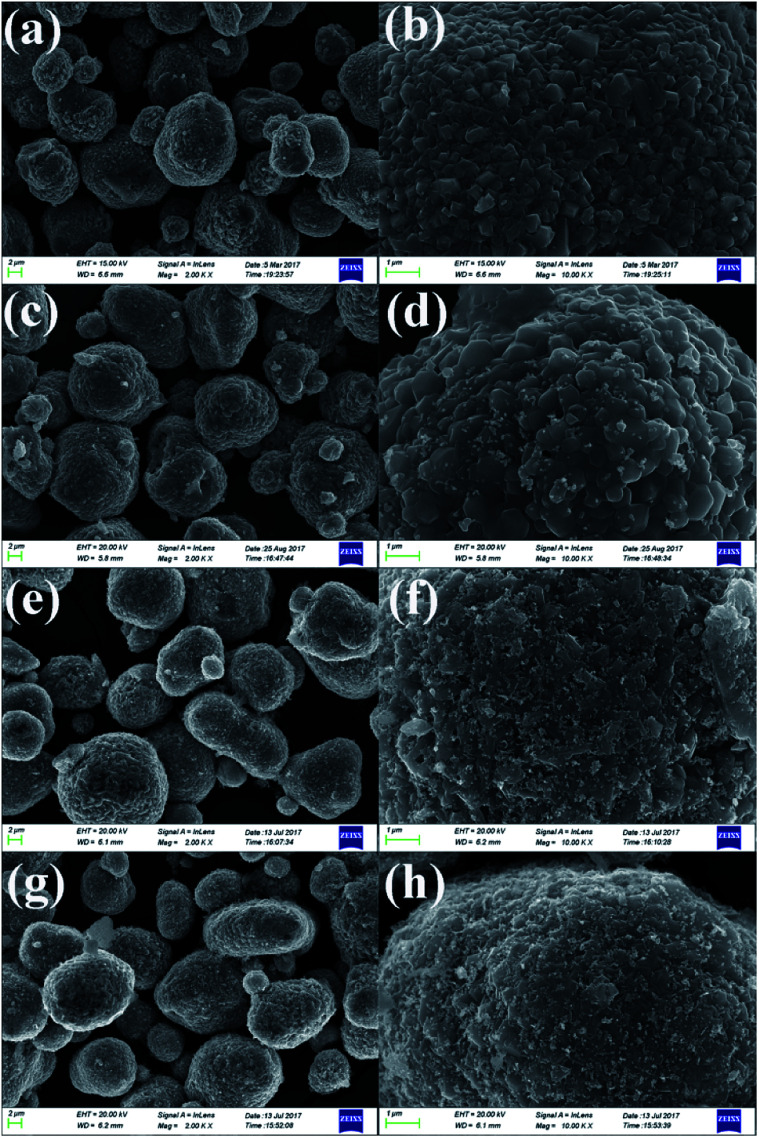
SEM images of (a and b) the pristine LiMn_2_O_4_, (c and d) 1 wt%, (e and f) 3 wt%, and (g and h) 5 wt% ZrO_2_-coated LiMn_2_O_4_ obtained by calcining 0, 1.19, 3.56, and 5.93 wt% ZrO_2_-coated Mn_3_O_4_ & Li_2_CO_3_ mixtures at 800 °C for 20 h.

As shown in Fig. 3a, c, e and g, low-magnification SEM images of the pristine and different amounts of ZrO_2_-coated LiMn_2_O_4_ materials heat-treated at 800 °C for 20 h (the coating material is ZrO_2_, which can be proven in the latter XRD data) illustrate that the spherical morphology is still maintained after long-term high-temperature heat-treatment. The average particle diameter is about 10 μm. Fig. 3b shows that the surface morphology of the pristine LiMn_2_O_4_ is smooth and clean. In contrast, the surfaces of the coated LiMn_2_O_4_ become rough and are covered with small particles, as shown in Fig. 3d, f and h. The coating layer becomes more and more obviously with the ZrO_2_ coating amount increasing from 1 to 5 wt%, which demonstrates that the coating layer is still maintained even after having been heat-treated at 800 °C for 20 h.

**Fig. 4:**
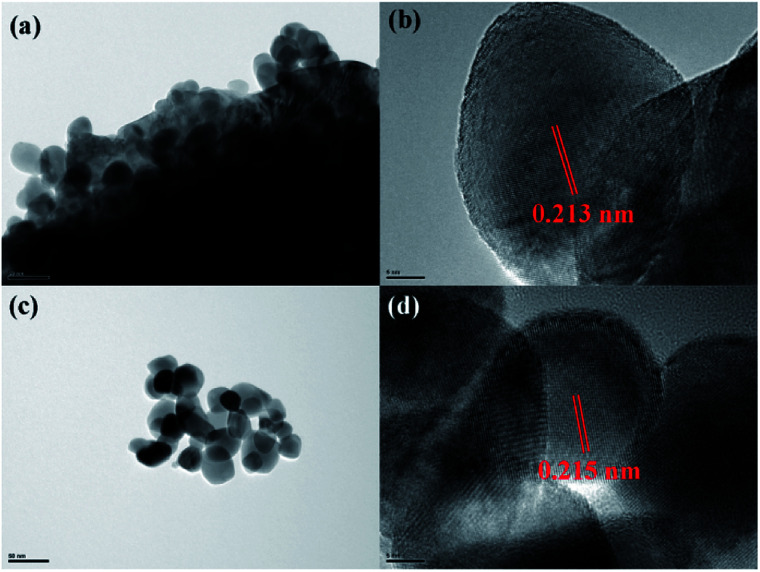
HRTEM images of (a and b) 3 wt% ZrO_2_-coated LiMn_2_O_4_ and (c and d) nano-sized ZrO_2_ heat-treated at 800 °C for 20 h.

Lattice structure of the coating layer on LiMn_2_O_4_ particles was investigated by HRTEM. As illustrated in Fig. 4a, HRTEM images show that there is a film-like layer as we speculate in SEM images. Fig. 4b and d show that the measured interplanar distances of the coating layer of 3 wt% ZrO_2_-coated LiMn_2_O_4_ and nano-sized ZrO_2_ heat-treated at 800 °C for 20 h are around 0.218 nm, respectively, which matches well to the (1̄21) plane of ZrO_2_ with monoclinic crystal system. Therefore, we can draw a conclusion that the coating layer on the surface of LiMn_2_O_4_ particles is ZrO_2_.

**Fig. 5:**
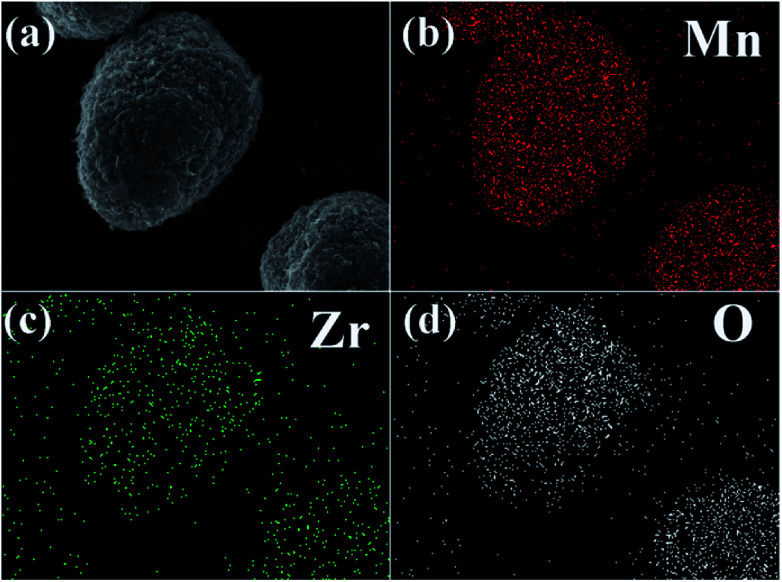
(a) SEM image of 3 wt% ZrO_2_-coated LiMn_2_O_4_ and the corresponding elemental mapping of (b) Mn, (c) Zr, and (d) O.

Fig. 5b–d shows elemental mapping studies on 3 wt% ZrO_2_-coated LiMn_2_O_4_ particles. It is clear that the presence of Mn, Zr, and O are homogenous within LiMn_2_O_4_ particles, and the ZrO_2_ coating layer is uniformly distributed on the surface of LiMn_2_O_4_ particles.

**Fig. 6:**
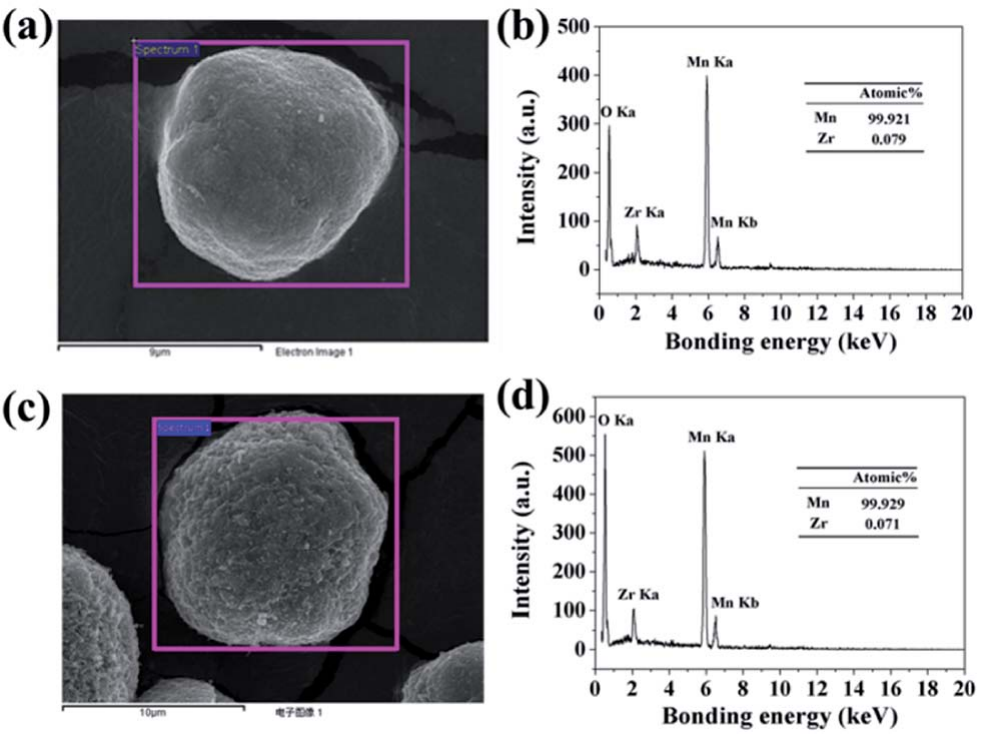
(a) SEM image of 3.56 wt% ZrO_2_-coated Mn_3_O_4_ and (b) its corresponding EDX spectroscopy. (c) SEM image of 3.0 wt% ZrO_2_-coated LiMn_2_O_4_ and (d) its corresponding EDX spectroscopy.

In order to confirm the extent of inter-diffusion between Zr and Mn ions in the process of high-temperature heat-treatment, EDX was used to measure the Zr/Mn atomic ratio in the particles before and after high-temperature calcination. As illustrated in Fig. 6b, the atomic ratio of Zr/Mn in 3.56 wt% ZrO_2_-coated Mn_3_O_4_ particle is 0.079/99.921. As to the final product of 3.0 wt% ZrO_2_-coated LiMn_2_O_4_ (Fig. 6d), the atomic ratio of Zr/Mn is 0.071/99.929, which is slightly lower than that of 3.56 wt% ZrO_2_-coated Mn_3_O_4_ particle. If all of Zr ions are diffused into the bulk LiMn_2_O_4_ particles, the atomic ratio of Zr/Mn would be 0.022/99.978. Therefore, we can draw a definite conclusion that the inter-diffusion between Zr and Mn ions is very little.

**Fig. 7:**
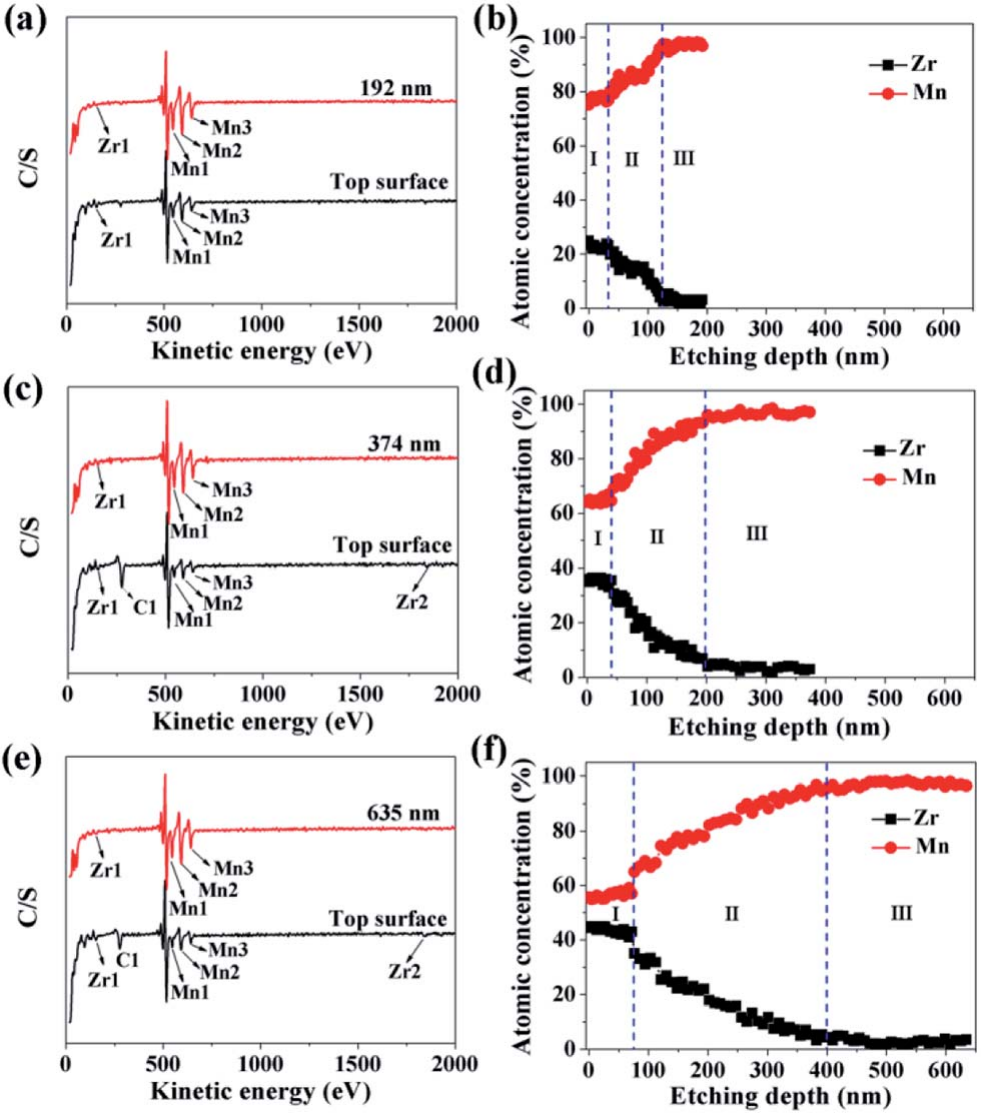
AES spectroscopies of (a) 1 wt%, (c) 3 wt%, and (e) 5 wt% ZrO_2_-coated LiMn_2_O_4_. Atomic concentration of Zr and Mn as a function of the etching depth: (b) 1 wt%, (d) 3 wt%, and (f) 5 wt% ZrO_2_-coated LiMn_2_O_4_.

In order to investigate the ZrO_2_ coating thickness, the theoretical value was firstly calculated. The calculation method can be seen in Fig. S3 in ESI.† The theoretical coating thickness of 1, 3, and 5 wt% ZrO_2_-coated LiMn_2_O_4_ materials is 12.2, 36.6, and 61.0 nm, respectively. AES was also employed to measure the actual coating thickness of different amounts of ZrO_2_-coated LiMn_2_O_4_ materials. From Fig. 7a, c and e, the peaks indexed to Zr1 can be easily observed on the top surface of 1, 3, and 5 wt% ZrO_2_-coated LiMn_2_O_4_ materials. As the Ar^+^ etching depth increases, the peak intensity of Zr1 decreases, while that of Mn1, Mn2, and Mn3 increases. When the etching depth reaches 192, 374, and 635 nm for 1, 3, and 5 wt% ZrO_2_-coated LiMn_2_O_4_ samples, though the Zr1 peak intensities are weak, they can still be observed, indicating that some Zr ions have diffused into the crystal lattice of LiMn_2_O_4_.

As illustrated in Fig. 7b, d and f, the changing trend of the atomic concentration of Zr and Mn can be divided into three stages: in the first stage (stage-I), the atomic concentration of Zr and Mn changes little, so the etching depth in stage-I can represent the thickness of ZrO_2_ coating layer. Therefore, it can be estimated from Fig. 7b, d and f that the coating thickness of 1, 3, and 5 wt% ZrO_2_-coated LiMn_2_O_4_ samples are 32, 42, and 75 nm, respectively. The measured thickness of the coating layer is slightly bigger than the theoretical value; the main reason is that the actual density of ZrO_2_ is reduced due to the nano-crystallization of particles. Since ZrO_2_ is a granular material, not a film material, so the surface coverage will be incomplete if the coating amount is small. As ZrO_2_ coating amount increases, the surface coverage becomes completely, and then the coating thickness will increase proportionally with the increase of ZrO_2_ coating amount. Considering that the coating thickness of 3 wt% ZrO_2_-coated LiMn_2_O_4_ sample is only 10 nm bigger than that of 1 wt% ZrO_2_-coated sample, and the ratio of the coating thickness between 5 wt% and 3 wt% coating amount is 1.786, which is very close to 5 : 3. So, it is speculated that the surface coverage for 1 wt% ZrO_2_ coating amount is not complete. As the ZrO_2_ coating amount is ≥3 wt%, the surface coverage becomes completely, which means that the “breakthrough” loading to have a complete coverage should be 3 wt%. In the second stage (stage-II), the atomic concentration of Zr drastically decreases and that of Mn rapidly increases, indicating that Zr ions have diffused into the crystal lattice in the surficial layer of LiMn_2_O_4_ particles to form a LiMn_2−*x*_Zr_*x*_O_4_ phase. According to Fig. 7b, d and f, the thickness of LiMn_2−*x*_Zr_*x*_O_4_ layer for 1, 3, and 5 wt% ZrO_2_-coated LiMn_2_O_4_ samples are estimated to be 92, 158, and 325 nm, respectively, indicating that more ZrO_2_ content makes more Zr ions diffuse into LiMn_2_O_4_ particles. In the third stage (stage-III), the atomic concentration of Zr is very low and changes very little with the etching depth increasing, indicating that it is very difficult for all of Zr ions to completely diffuse into the crystal lattice of LiMn_2_O_4_ particles. From the atomic concentration of Zr in the inner part of LiMn_2_O_4_ particles, we can obtain that some Zr ions have been doped to form a LiMn_2−*x*_Zr_*x*_O_4_ phase (0.01 ≤ *x* ≤ 0.02).

The SEM, HRTEM, EDX, and AES results show that the LiMn_2_O_4_ particles can be successfully coated by ZrO_2_*via* the one-time sintering process. This coating method is very easy to be scaled up and can be used to prepare other coating-type cathode materials. However, the successful synthesis of ZrO_2_-coated LiMn_2_O_4_*via* the one-time sintering process is just a specific example. In order to make this method more universal, we systematically studied what factors are essential to guarantee its success.

First of all, the blending manner of raw materials may be one of the key factors. In the preceding experiments, the Mn_3_O_4_ precursor was coated with nano-sized ZrO_2_ in advance, and then Li_2_CO_3_ was added and further ball-milled for 3 h. Herein, we made an adjustment to the blending manner as below. Three raw materials of Mn_3_O_4_, Li_2_CO_3_, and ZrO_2_ (the amount of ZrO_2_ is 3.56 wt% of the weight of Mn_3_O_4_) were ball-milled simultaneously, and then the mixture was preheated at 550 °C for 5 h and then calcined at 800 °C for 20 h. The as-prepared material is denoted as LMO@ZrO_2_ and deemed as a control sample. SEM images of LMO@ZrO_2_ are illustrated in Fig. S4(a and b).† It can be seen that there is very small amount of ZrO_2_ observed on the surface of the LiMn_2_O_4_ particles. However, as illustrated in Fig. S4(c and d),† a large number of ZrO_2_ particles can be successfully coated onto the LiMn_2_O_4_ particles through calcining the mixture of Li_2_CO_3_ and ZrO_2_-coated Mn_3_O_4_. According to the above comparison results, a definite conclusion can be drawn: only the precursor is coated with the coating material beforehand, can the coating material be uniformly coated onto the surface of the final particles *via* the one-time sintering process. Besides, it can also be seen from Fig. S4(e and f)† that the coating uniformity of the one-time sintering process is similar to that of the conventional coating method.

**Fig. 8:**
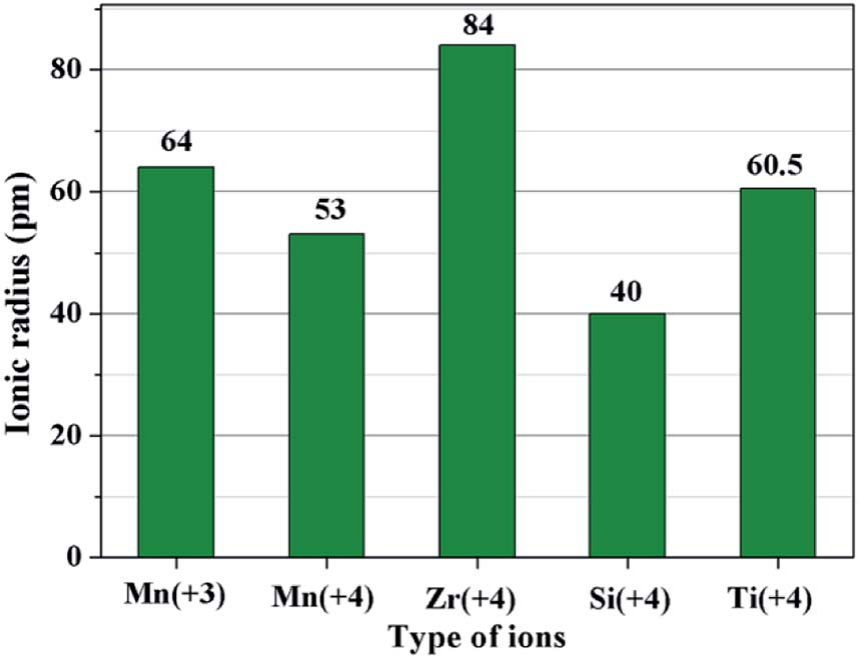
The ionic radii of five types of ions: Mn^3+^, Mn^4+^, Zr^4+^, Si^4+^, and Ti^4+^.

The second key factor may be choosing a suitable coating material. According to the diffusion theory, among the various physicochemical properties of the coating materials, the ionic radius may play an important role.^32^ In order to prove the above assumption, two other metal ions of Ti^4+^ and Si^4+^ are chosen to synthesize different amounts of SiO_2_- and TiO_2_-coated LiMn_2_O_4_ materials *via* the one-time sintering process. Unexpectedly, the result obtained from Fig. S6–S9† demonstrates that SiO_2_- and TiO_2_-coated LiMn_2_O_4_ materials cannot be successfully synthesized by the one-time sintering process, which indicates that it is easy for Si^4+^ and Ti^4+^ to diffuse into the bulk LiMn_2_O_4_ crystals at 800 °C. In order to compare the ionic radius difference among Mn^3+^, Mn^4+^, Zr^4+^, Si^4+^, and Ti^4+^, their ionic radii are shown in Fig. 8. It is seen that Zr^4+^ has a much larger ionic radius than Mn^3+^ and Mn^4+^. While, the ionic radius of Si^4+^ is much smaller than those of Mn^3+^ and Mn^4+^, and the ionic radius of Ti^4+^ is between those of Mn^3+^ and Mn^4+^. From the comparable results of ZrO_2_, SiO_2_, and TiO_2_ coating layers after heat-treatment at 800 °C, we can conclude that the successful synthesis of ZrO_2_-coated LiMn_2_O_4_*via* the one-time sintering process is probably due to the much larger ionic radius of Zr^4+^ than those of Mn^3+^ and Mn^4+^. Therefore, the ionic radius is also a crucial factor to realize the coating-type materials *via* the one-time sintering process.

**Fig. 9:**
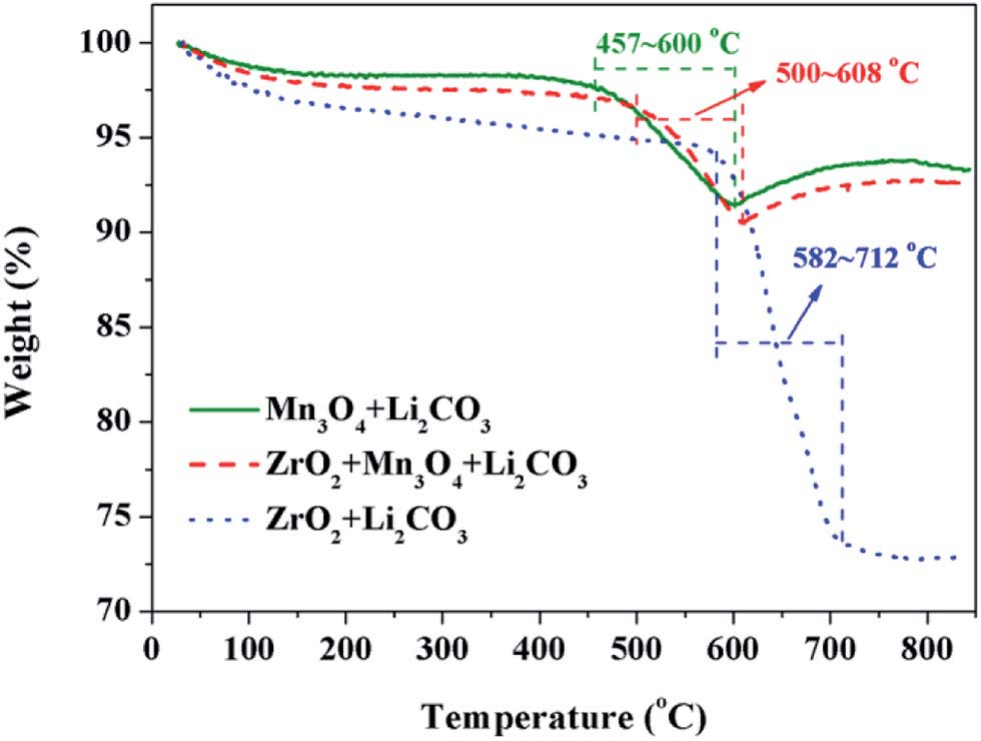
TG curves of the raw material mixtures with a heating rate of 3 °C min^−1^ in air.

The third key factor should be the choice of the pre-calcination temperature. If the reaction between Li_2_CO_3_ and ZrO_2_ is ahead of that Li_2_CO_3_ and Mn_3_O_4_, the coating material may be Li_2_ZrO_3_ and not ZrO_2_, thus some extra Li_2_CO_3_ will be consumed in vain and the loss of the specific discharge capacity of LiMn_2_O_4_ will be more seriously. In order to choose a suitable pre-calcination temperature, TG-DTA was performed to reveal the reaction process between Li_2_CO_3_ and ZrO_2_-coated Mn_3_O_4_ and the result is shown in Fig. 9. For the mixture of Li_2_CO_3_ and Mn_3_O_4_, the reaction temperature range is between 457 °C and 600 °C. While for the mixture of Li_2_CO_3_ and ZrO_2_, the reaction temperature range is between 582 °C and 712 °C. Besides, it is also noticed that when the Mn_3_O_4_ is coated with ZrO_2_ beforehand, the starting reaction temperature between Li_2_CO_3_ and Mn_3_O_4_ is postponed by 43 °C to 500 °C. This is because Li^+^ must pass through the ZrO_2_ coating layer and then can react with Mn_3_O_4_ to form LiMn_2_O_4_. Although there is overlap in the reaction temperature range between the mixtures of Li_2_CO_3_ & ZrO_2_-coated Mn_3_O_4_ and Li_2_CO_3_ & ZrO_2_, it is probably that Li_2_CO_3_ would react only with Mn_3_O_4_ and not with ZrO_2_ if the pre-calcination temperature is set at 550 °C.

Influence of the sintering temperature and time on the crystal structure and performance of the pristine LiMn_2_O_4_ and different amounts of ZrO_2_-coated LiMn_2_O_4_ materials were investigated in detail. As illustrated in Fig. S10 and S11,† the diffraction peaks of ZrO_2_ can always be observed between 550 °C to 850 °C, which shows that the coating layer is indeed ZrO_2_ and the coating layers do not disappear even after long-term high-temperature calcination. Fig. S12† shows the influence of sintering temperature on cyclic performance of the pristine LiMn_2_O_4_, and the optimal synthesizing temperature is 800 °C. Fig. S13† demonstrates that the discharge capacity slightly increases and the capacity retention slightly decreases with the sintering time increasing. Therefore, the synthesizing condition of the pristine LiMn_2_O_4_ and different amounts of ZrO_2_-coated LiMn_2_O_4_ materials is set at 800 °C for 20 h.

**Fig. 10:**
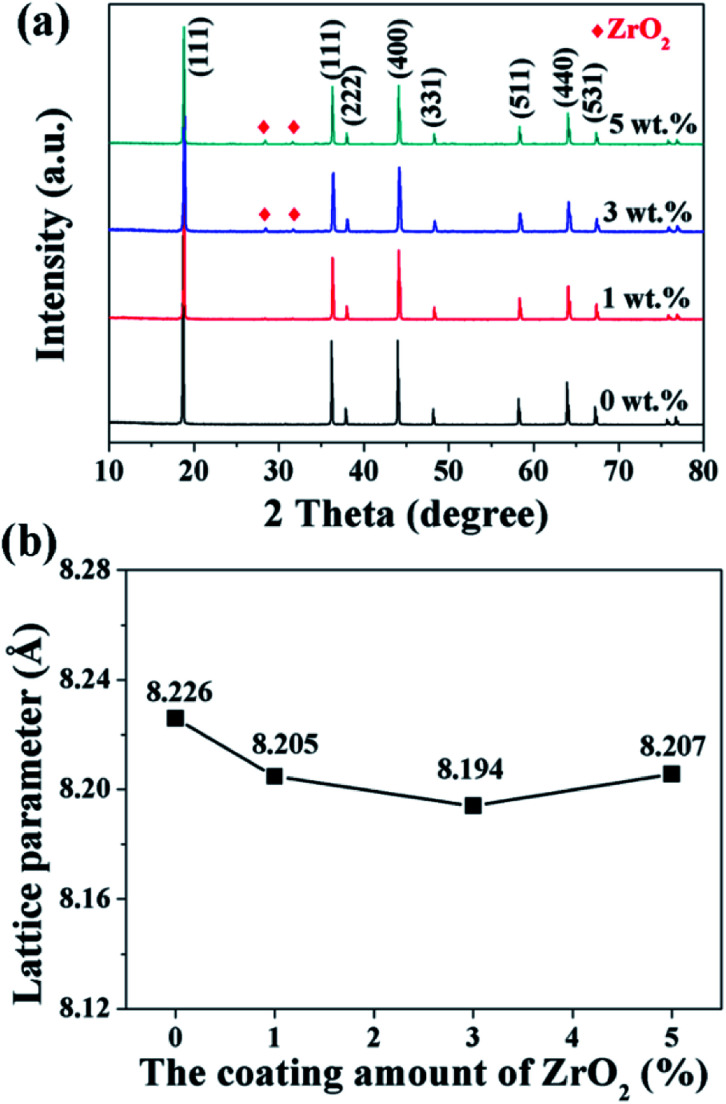
(a) XRD patterns and (b) the lattice parameters of 1, 3, and 5 wt% ZrO_2_-coated LiMn_2_O_4_ materials.

The XRD patterns of the pristine and different amounts of ZrO_2_-coated LiMn_2_O_4_ materials calcined at 800 °C for 20 h are shown in Fig. 10a. The major diffraction peaks of the pristine LiMn_2_O_4_ are in good agreement with that obtained from JCPDF file no. 35-0782, corresponding to the cubic spinel structure with *Fd*3̄*m* space group. For the 1 wt% ZrO_2_-coated LiMn_2_O_4_ sample, there is no diffraction peaks corresponding to ZrO_2_ observed in the XRD pattern, this is because the ZrO_2_ amount is too low to be detected. As expected, the impurity phase of ZrO_2_ is obviously observed with the coating amount increasing to 3 wt% and 5 wt%, which demonstrates that most of ZrO_2_ do not react with Li_2_CO_3_. A suitable pre-calcination temperature can guarantee that Li_2_CO_3_ does not react with ZrO_2_ to form Li_2_ZrO_3_, which leads to two advantages as below: (i) the weight of the coating layer is not further increased, which will not greatly reduce the specific discharge capacity of the final products; (ii) extra Li_2_CO_3_ won't be consumed in vain. Therefore, the suitable pre-calcination temperature is the key factor to determine the chemical compositions of the coating materials.

Furthermore, the lattice parameter *a* (= *b* = *c*) is also calculated from the XRD data and the results are shown in Fig. 10b. It is interesting that the lattice parameter of ZrO_2_-coated LiMn_2_O_4_ materials is slightly smaller than that of the pristine LiMn_2_O_4_ material. The reason is probably that after heat-treatment at such high temperature of 800 °C for 20 h, a small fraction of Zr^4+^ diffuses into the spinel lattice of the bulk LiMn_2_O_4_ to partial substitute the site of Mn^4+^. Ionic radius of Zr^4+^ is much bigger than that of Mn^4+^, the lattice constant should be bigger if Zr^4+^ ions substitute the site of Mn^4+^ in the spinel. But the fact is on the contrary. Actually, the lattice constant of spinel LiMn_2_O_4_ crystal is not only related to the ionic radius, but also related to the M–O bond energy.^33^ The Δ_f_*G*^Θ^(ZrO_2_) and Δ_f_*G*^Θ^(MnO_2_) is −1042.8 kJ mol^−1^ and −465.1 kJ mol^−1^, respectively. The Δ_f_*G*^Θ^(ZrO_2_) is much more negative than Δ_f_*G*^Θ^(MnO_2_), which can deduce that bond energy of Zr–O is much stronger than Mn–O and the crystal volume shrinks. Therefore, the structure with Zr^4+^ doping is more compact and has smaller lattice constant. Besides, the lattice parameter changes little with ZrO_2_ content increasing from 1 wt% to 5 wt%, the reason is that the doping content of Zr^4+^ in the inner part of LiMn_2_O_4_ is almost constant, no matter the ZrO_2_ coating amount is 1 wt%, 3 wt% or 5 wt%. It implies that the upper limit of the actual Zr^4+^ amounts diffusing into the spinel lattice of LiMn_2_O_4_ is very small, primarily because the ionic radius of Zr^4+^ is much larger than that of Mn^4+^.

**Fig. 11:**
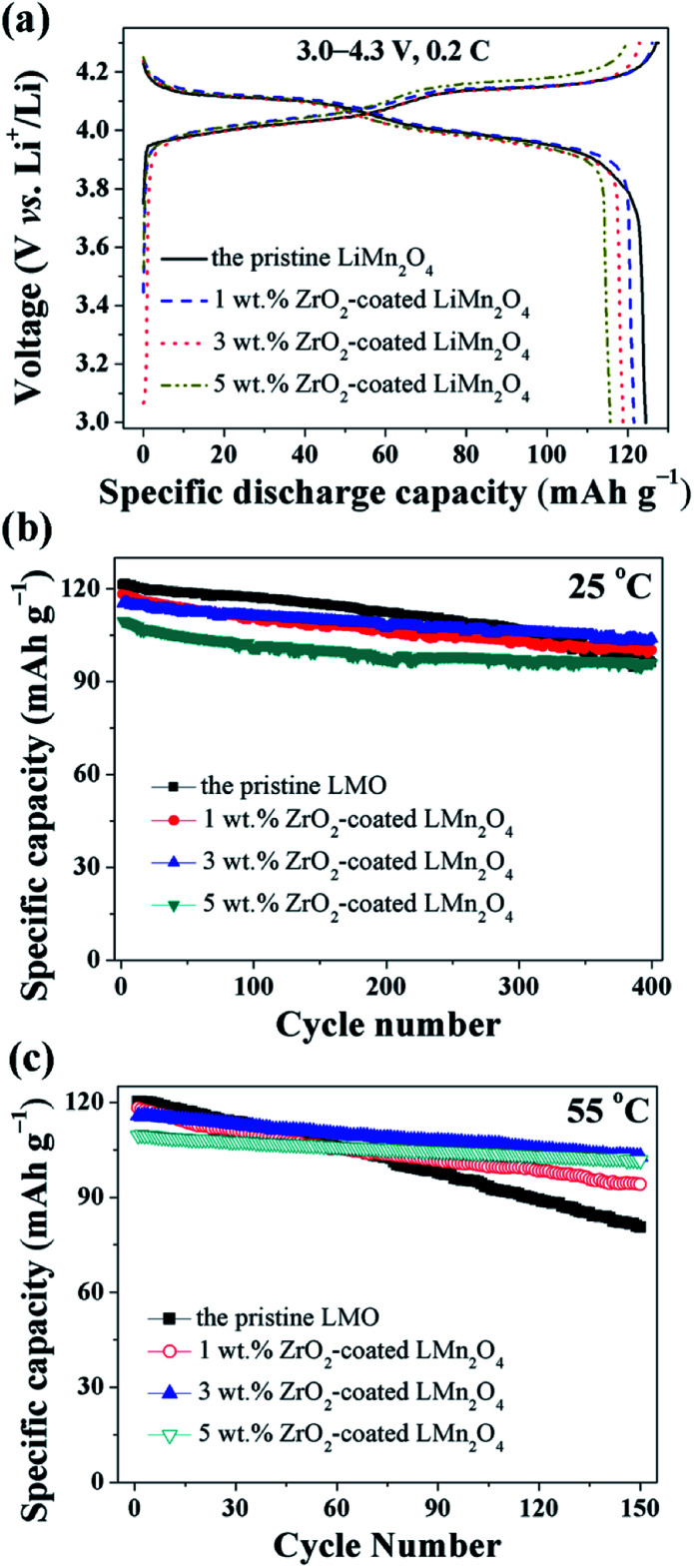
(a) The charge–discharge curves of 1, 3, and 5 wt% ZrO_2_-coated LiMn_2_O_4_ materials with the current rate of 0.2C. The cycling performance of 1, 3, and 5 wt% ZrO_2_-coated LiMn_2_O_4_ materials tested at (b) 25 °C and (c) 55 °C at 1C.

The electrochemical properties of the pristine LiMn_2_O_4_ and different amounts of ZrO_2_-coated LiMn_2_O_4_ materials were studied in the voltage range of 3.0–4.3 V at 25 °C and 55 °C. Fig. 11a shows the initial charge–discharge curves of different amounts of ZrO_2_-coated LiMn_2_O_4_ materials at a current rate of 24 mA g^−1^ corresponding to 0.2C. It can be seen that all discharge curves have two voltage plateaus at approximately 3.95 V and 4.1 V, which is typical for LiMn_2_O_4_ and its variants. The two voltage plateaus indicate that the extraction (and subsequent re-insertion) of lithium ions from tetrahedral sites occurs in two stages. The pristine LiMn_2_O_4_ material delivers the highest specific discharge capacity of 124.4 mA h g^−1^ with the initial columbic efficiency of 97.6%. The specific discharge capacities of ZrO_2_-coated LiMn_2_O_4_ materials decrease with the ZrO_2_ coating amount increasing. The initial discharge capacity of 1, 3, and 5 wt% ZrO_2_-coated LiMn_2_O_4_ is 121.5, 118.8, and 115.6 mA h g^−1^ with the initial columbic efficiency of 96.4, 96.7, and 96.1%, respectively. The RT and HT cycling performance of the pristine and different amounts of ZrO_2_-coated LiMn_2_O_4_ materials is evaluated with the current density of 120 mA g^−1^ corresponding to 1C and the results are shown in Fig. 11b and c. At 25 °C, the initial specific discharge capacity of 0, 1, 3, and 5 wt% ZrO_2_-coated LiMn_2_O_4_ materials is 121.3, 118.3, 115.2, and 109.6 mA h g^−1^ with the 400^th^ capacity retention of 79.2, 84.7, 90.1, and 87.5%, respectively. As the test temperature increases to 55 °C, the pristine LiMn_2_O_4_ shows severe capacity loss with the capacity retention of 67.0% after 150 cycles. While the specific discharge capacity of 1, 3, and 5 wt% ZrO_2_-coated LiMn_2_O_4_ after 150 cycles is 94.1, 102.8, and 101.7 mA h g^−1^ with the capacity retention of 79.5, 88.9, and 92.9%, respectively. Compared with the pristine LiMn_2_O_4_, though the specific discharge capacities of ZrO_2_-coated LiMn_2_O_4_ decrease, their cycling performance is greatly improved. By comparison of the comprehensive electrochemical performance, the 3 wt% coating amount is preferred, because it has a relatively high specific discharge capacity and an excellent cycling performance.

**Fig. 12:**
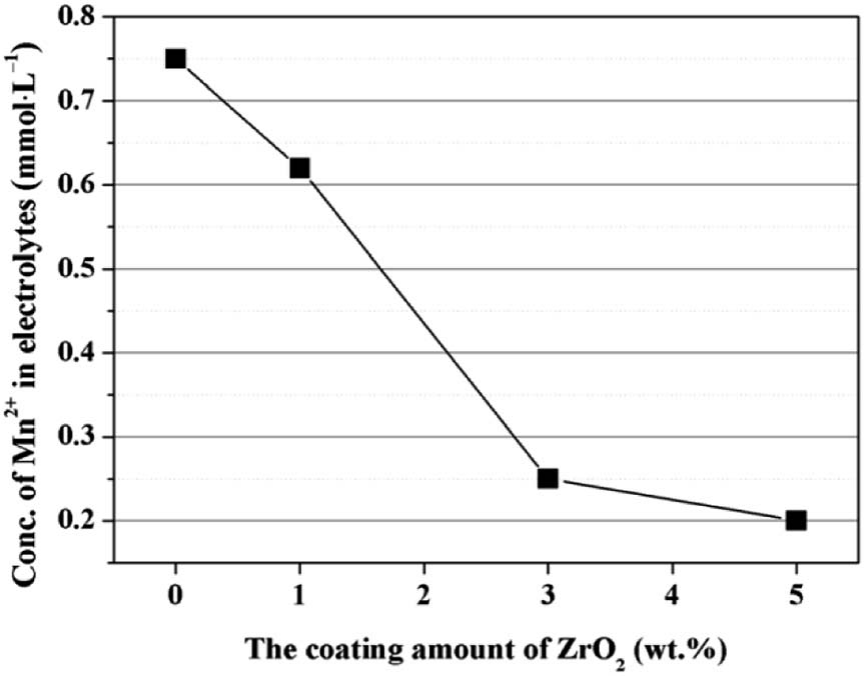
Concentration of Mn^2+^ in the electrolyte as a function of the coating amounts of ZrO_2_.

The enhanced cycling performance should be mainly contributed to the ZrO_2_ coating layer, which can suppress the Mn^2+^ dissolution from LiMn_2_O_4_ cathode into the electrolyte. In order to measure the Mn^2+^ dissolution, 0.3 g cathode materials were added into 10 mL electrolyte and stored for 7 days at 60 °C, and then the Mn^2+^ concentration was tested by ICP. As illustrated in Fig. 12, the Mn^2+^ concentration in electrolyte for the pristine LiMn_2_O_4_ is 0.75 mmol L^−1^. In comparison, the Mn^2+^ concentrations in electrolyte for 1, 3, and 5 wt% ZrO_2_-coated LiMn_2_O_4_ materials drops quickly to 0.62, 0.25, and 0.2 mmol L^−1^, which proves that the ZrO_2_ coating layer can greatly inhibit the Mn^2+^ dissolution from the active material into the electrolyte.

In order to investigate the stability of the ZrO_2_ coating layer, SEM and XRD were used to characterize the morphology and crystal structure of 3 wt% ZrO_2_-coated LiMn_2_O_4_ electrode post electrochemical testing, and the results are shown in Fig. S14 and S15.† Based on the analysis of SEM and XRD data, we can conclude that the ZrO_2_ coating layer on the surface of LiMn_2_O_4_ particles is very stable to the volume changes brought by the long-term charge–discharge processes. The reason is probably that the coating layer calcined at 800 °C is very rigid and stable.

## Conclusions

In summary, different amounts of ZrO_2_-coated LiMn_2_O_4_ materials are successfully synthesized *via* a one-time sintering process. Three key factors to realize ZrO_2_-coated LiMn_2_O_4_ materials are as follows: (i) the Mn_3_O_4_ precursor is coated by nano-sized ZrO_2_ in advance; (ii) the ionic radius of Zr^4+^ is much larger than those of Mn^3+^ and Mn^4+^; (iii) the pre-calcination temperature is set in the reaction temperature range between Li_2_CO_3_ and Mn_3_O_4_ and lower than that between Li_2_CO_3_ and ZrO_2_. The as-prepared 3 wt% ZrO_2_-coated LiMn_2_O_4_ material exhibits an excellent electrochemical performance with the initial specific discharge capacity of 118.8 mA h g^−1^ at 0.2C and the capacity retention of 90.1% after 400 cycles at 25 °C and 88.9% after 150 cycles at 55 °C at 1C. The enhancement of the cycling performance is mainly contributed to the ZrO_2_ coating layer which can suppress the side reactions between LiMn_2_O_4_ and the electrolyte. Most significantly, the one-time sintering process to synthesize ZrO_2_-coated LiMn_2_O_4_ materials is very simple, low-cost, environmental friendly, and easy for large-scale industrial production, so as to promote its practical application and provide a valuable reference for synthesizing other coating-type cathode materials for LIBs.

## Conflicts of interest

There are no conflicts to declare.

## Supplementary Material

RA-008-C8RA01421C-s001
