# Supplementary material for: One-time sintering process to synthesize ZrO2-coated LiMn2O4 materials for lithium-ion batteries
Source: RSC Adv. 2018 May 8;8(30):16753–61. doi: 10.1039/c8ra01421c (PMC9080295; doi:10.1039/c8ra01421c)
Supplement: RA-008-C8RA01421C-s001 [file RA-008-C8RA01421C-s001.pdf]

Electronic Supplementary Material (ESI) for RSC Advances.
This journal is © The Royal Society of Chemistry 2018

Supporting information

One-time sintering process to synthesize ZrO_2-coated $LiMn_2O_4$ materials for lithium-ion batteries

Gang Li[a], Xu Chen[a], Yafei Liu[b], Yanbin Chen[b] and Wensheng Yang[a]*

[a] *State Key Laboratory of Chemical Resource Engineering, Beijing University of Chemical Technology, Beijing 100029, P.R. China*

[b] *Beijing Easpring Material Technology Co., Ltd., Beijing 100160, P. R. China*

Corresponding author: W. Yang, yangws@mail.buct.edu.cn

Tel.: +86 10 64435271; fax: +86 10 64425385

1. SEM and particle size distribution characterizations of raw materials

Mn_3O_4 (Industrial grade, Nanjing Tianyuan Magnetic Material Co., Ltd.) and ZrO_2 (Industrial grade, Xuan Cheng Jing Rui New Material Co., Ltd.) were used as the raw materials without any further treatment.

Particle size distribution of Mn_3O_4 and ZrO_2 was measured using the Mastersizer 2000 system. As illustrated in Fig. S1, the average particle size (D50) of Mn_3O_4 and ZrO_2 are 10.12 and 1.627 μm, respectively. Due to the aggregation of nano-sized ZrO_2, the measured value is much larger than the actual value.

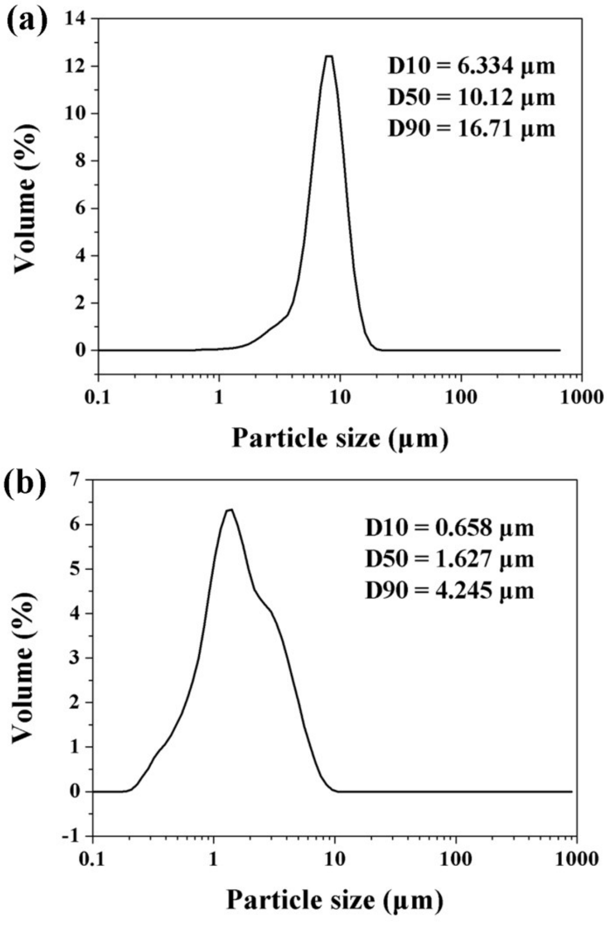

Fig. S1. Particle size distributions of (a) Mn_3O_4 and (b) ZrO_2.

SEM images of the raw materials of Mn_3O_4 and ZrO_2 were illustrated in Fig. S2. In can be seen from Fig. S2 that Mn_3O_4 particles have spherical-like appearance and are composed of the octahedral primary particles with the particle size ranging from 100 to 200 nm. Nano-sized ZrO_2 particles with the primary particle size of 30~40 nm aggregate into irregular secondary particles.

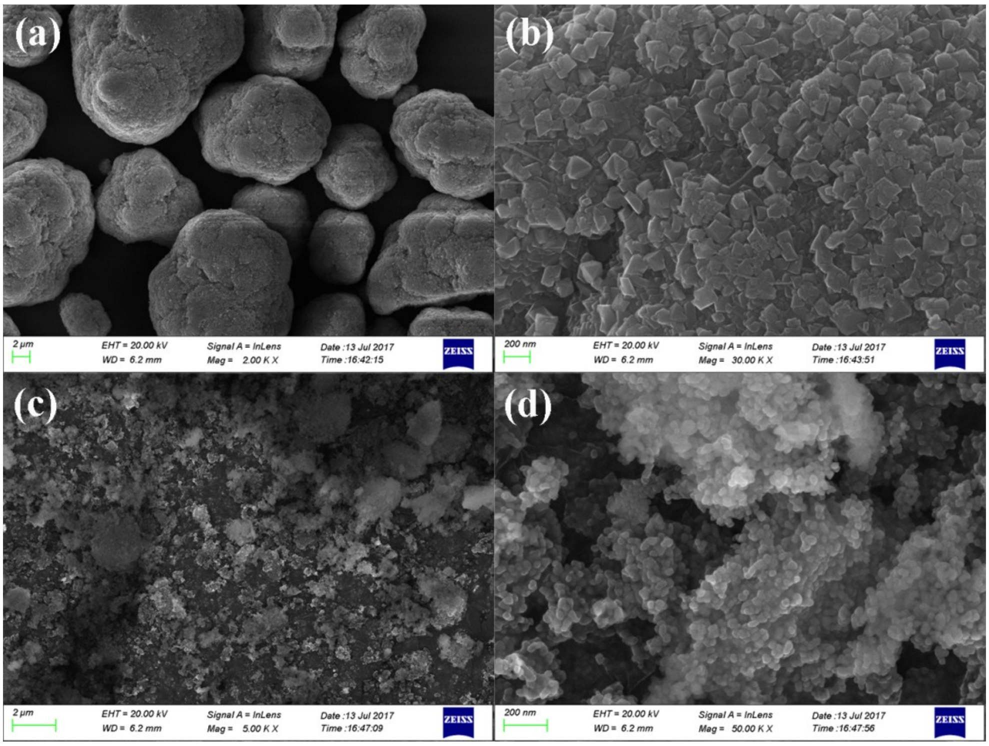

Fig. S2. SEM images of (a, b) Mn_3O_4 and (c, d) ZrO_2.

2. Material preparation

2.1. Synthesis of LMO@ZrO_2

Stoichiometric amounts of industrial grade Mn_3O_4, battery grade Li_2CO_3, and nano-sized ZrO_2 (Li:Mn=1.05:2) are accurately weighted and then ball-milled at the same time for 3 h (the adding amount of ZrO_2 is 3.56 wt.% of the weight of the Mn_3O_4 precursor). The mass ratio of the agate balls to the raw materials is 1:1, and the rotational speed of the planetary ball mill is 400 rpm. The obtained mixture was added into alumina crucible, then preheated at 550 ^oC for 5 h and calcined at 800 ^oC for 20 h in O_2 atmosphere. Both of the heating and cooling rates are 3 $^oC{\cdot}min^{-1}$. The calcined samples were crushed down and sifted through a sieve of 325 meshes, thus the final products were obtained. The as-synthesized material is denoted as LMO@ZrO_2.

2.2. Synthesis of 3 wt.% ZrO_2-coated $LiMn_2O_4$ material by dry coating method

3 wt.% ZrO_2-coated $LiMn_2O_4$ material was synthesized by the conventional dry coating method. $LiMn_2O_4$ was firstly synthesized by sintering the mixture of Mn_3O_4 and Li_2CO_3 (Li:Mn=1.05:2, molar ratio) at 800 ^oC for 20 h. Then, $LiMn_2O_4$ and nano-sized ZrO_2 materials were added into the ball-milling jar, ball-milled for 1 h, and followed by re-calcination at 600 ^oC for 5 h.

2.3. Synthesis of different amounts of SiO_2-coated $LiMn_2O_4$ materials

Industrial grade Mn_3O_4 and nano-sized SiO_2 were firstly weighed and ball-milled for 1 h, and then the stoichiometric amount of battery grade Li_2CO_3 (Li:Mn=1.05:2, molar ratio) was added to the above mixed materials and further ball-milled for 3 h. The mixture was preheated at 550 ^oC for 5 h and calcined at 800 ^oC for 20 h in O_2 atmosphere. Both of the heating and cooling rates are 3 $^oC{\cdot}min^{-1}$. Note that the adding amount of SiO_2 was calculated based on the weight of the final $LiMn_2O_4$ material. Therefore, in order to synthesize 1, 3, and 5 wt.% SiO_2-coated $LiMn_2O_4$, the adding amount of SiO_2 is 1.19, 3.56, and 5.93 wt.% of the weight of the Mn_3O_4 precursor.

2.4. Synthesis of different amounts of TiO_2-coated $LiMn_2O_4$ materials

Industrial grade Mn_3O_4 and nano-sized TiO_2 were firstly weighed and ball-milled for 1 h, and then the stoichiometric amount of battery grade Li_2CO_3 (Li:Mn=1.05:2, molar ratio) was

added to the above mixed materials and further ball-milled for 3 h. The mixture was preheated at 550 ^{o}C for 5 h and calcined at 800 ^{o}C for 20 h in O_2 atmosphere, then slowly cooled to 300 ^{o}C at a rate of 3 $^{o}C \cdot min^{-1}$. Note that the adding amount of TiO_2 was calculated based on the weight of the final $LiMn_2O_4$ material. Therefore, in order to synthesize 1, 3, and 5 wt.% TiO_2-coated $LiMn_2O_4$, the adding amount of TiO_2 is 1.19, 3.56, and 5.93 wt.% of the weight of the Mn_3O_4 precursor.

3. The theoretical calculation of the coating thickness

The calculation model for the coating thickness on the particle surface of $LiMn_2O_4$ is shown in Fig. S3. D is the average particle size of spherical $LiMn_2O_4$ materials, and d(ZrO_2) is the coating thickness of ZrO_2.

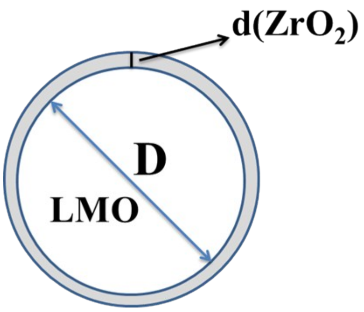

Fig. S3 The calculation model for the coating thickness of ZrO_2 on the particle surface of $LiMn_2O_4$.

Now, we define the volume, mass, and surface area of a single spherical $LiMn_2O_4$ as V(SLMO), m(SLMO), and S(SLMO), respectively. (1 wt.% ZrO_2-coated $LiMn_2O_4$ as an example)

The true densities of $LiMn_2O_4$ and ZrO_2 are 4.28 and 5.85 $g \cdot cm^{-3}$, respectively. The average particle size of $LiMn_2O_4$ is ~10 μm.

$V(SLMO) = (4\pi r^3)/3 = (4\times3.14\times5^3)/3 = 523.3\ (\mu m^3)$

$S(SLMO) = \pi D^2 = 3.14\times 10^2 = 314\ \mu m^2$

$m(SLMO) = \rho V = 4.28 \times 10^{-12} \times 523.3 = 2.24 \times 10^{-9}\ (g)$

$m(ZrO_2) = 2.24 \times 10^{-9} \times 1\% = 2.24 \times 10^{-11}\ (g)$

Because the coating thickness is very small, so the volume of ZrO_2 can be calculated as below:

$V(ZrO_2) = S(SLMO) \cdot d(ZrO_2)$

So, $m(ZrO_2) = S(SLMO) \cdot d(ZrO_2) \cdot \rho(ZrO_2)$

$= 2.24\times 10^{-11} \div (3.14 \times 10^2 \times 10^6) \div (5.85 \times 10^{21})$

$= 12.2\ (nm)$

Based on the above formula, the theoretical coating thickness of 1, 3, and 5 wt.% ZrO_2-coated $LiMn_2O_4$ materials is 12.2, 36.6, and 61.0 nm, respectively.

4. Effect of the blending method on the coating uniformity of the final materials

SEM images of LMO@ZrO_2 are shown in Fig. S4(a, b). It can be seen from Fig. S4(a, b) that there are very small amount of ZrO_2 particles on the surface of LMO@ZrO_2 particles. The reason is that three raw materials are ball-milled at the same time, which makes a great number of ZrO_2 particles attached to the surface of Li_2CO_3 particles. Therefore, even though Li_2CO_3 would react with Mn_3O_4 to form $LiMn_2O_4$ after being heat-treated at 800 ^{o}C for 20 h, the ZrO_2 on the surface of Li_2CO_3 particles can't be coated onto the surface of the final $LiMn_2O_4$ particles again. So, the coating uniformity of LMO@ZrO_2 is very poor. By contrast, as illustrated in Fig. S4(c, d), using the two-step mixing method, the coating layer of 3 wt.% ZrO_2-coated $LiMn_2O_4$ synthesized via the one-time sintering process is very uniform. This is because the Mn_3O_4 precursor is coated with ZrO_2 beforehand, which can guarantee that ZrO_2 particles would not be coated on the surface of Li_2CO_3 particles or exist alone. Thus, a great majority of ZrO_2 particles can be coated on the final $LiMn_2O_4$ particles after high-temperature calcination.

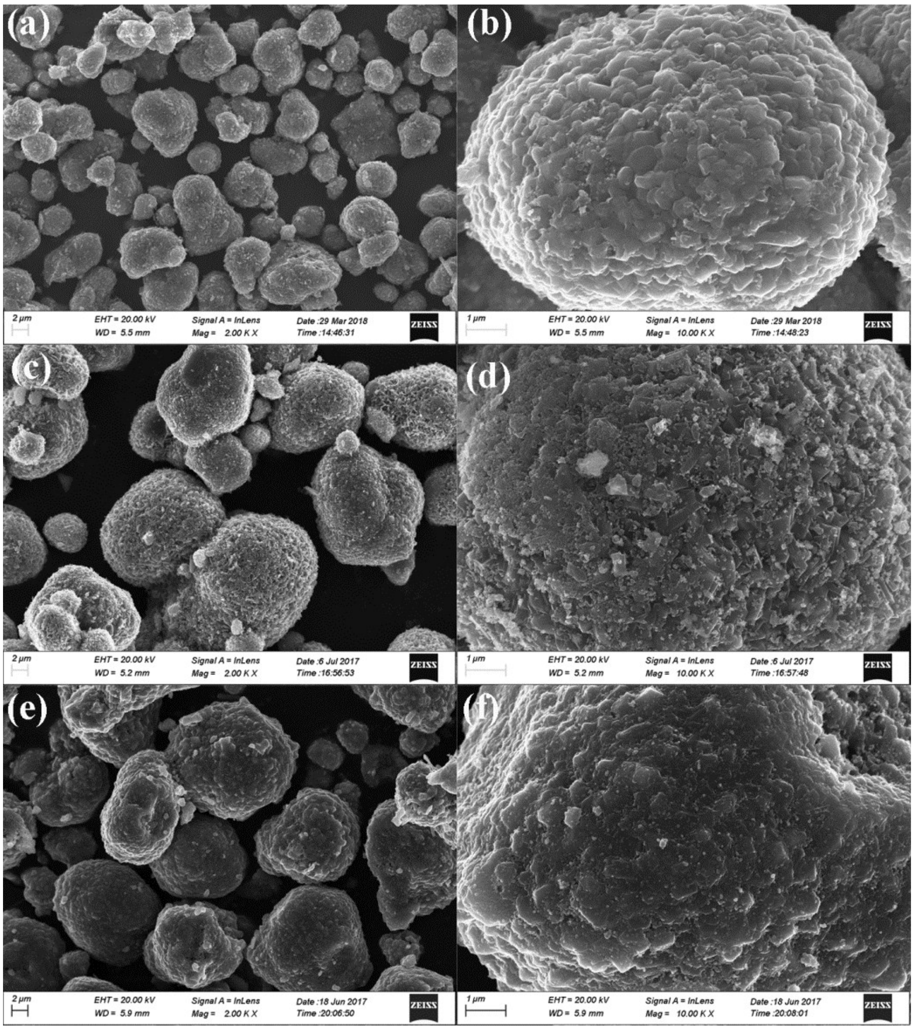

Fig. S4. SEM images of (a, b) LMO@ZrO_2 material, (c, d) 3 ZrO_2-coated $LiMn_2O_4$ synthesized via one-time sintering process, and (e, f) 3 ZrO_2-coated $LiMn_2O_4$ synthesized by the conventional dry coating method.

The cyclic performances of the pristine $LiMn_2O_4$ and 3 wt.% ZrO_2-coated $LiMn_2O_4$ materials synthesized by the one-time sintering process and the conventional dry coating process are investigated at 25 ^oC and 55 ^oC. Their electrochemical properties are shown in Fig. S5 and Table S1. From Table S1, both of the room-temperature (RT) and high-temperature (HT) cyclic performance for 3 wt.% ZrO_2-coated $LiMn_2O_4$ synthesized by two different coating methods are greatly improved. In contrast, the initial discharge capacity of the sample synthesized by one-time sintering process is slightly lower than that of the sample synthesized by the conventional coating method, but it is opposite as to the cyclic performance. The main reasons are as follows: For the one-time sintering coating method, a small portion of Zr ions diffuse into the inner part of $LiMn_2O_4$ particles, thus the electrochemical active Mn^{3+} content is reduced and the spinel structure stabilization is increased. For the conventional coating

method, due to the relatively lower sintering temperature of 600 °C, ZrO_2 only plays a role of coating effect.

In summary, the one-time sintering process to synthesize ZrO_2-coated $LiMn_2O_4$ can exhibit a similar electrochemial performance with the conventional coating method.

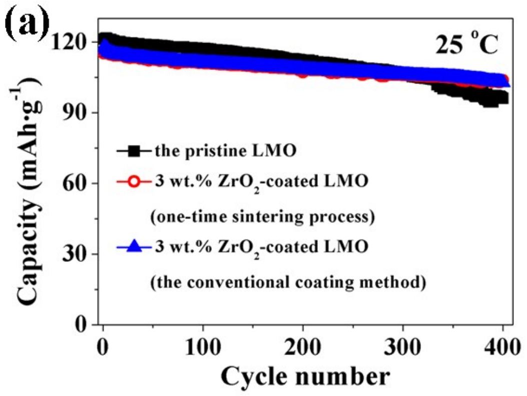

Fig. S5. Cyclic performance of the pristine $LiMn_2O_4$ and 3 wt.% ZrO_2-coated $LiMn_2O_4$ synthesized by one-time sintering process and the conventional coating method.

Table S1. The electrochemical properties of the pristine $LiMn_2O_4$ (sample-1) and 3 wt.% ZrO_2-coated $LiMn_2O_4$ synthesized by one-time sintering process (sample-2) and the conventional coating method (sample-3).

Sample	25 °C		55 °C	
	Initial capacity (mAh·g^{-1})	400th capacity retention (%)	Initial capacity (mAh·g^{-1})	150th capacity retention (%)
sample-1	121.3	79.2	121.5	67.0
sample-2	115.2	90.1	115.7	88.9
sample-3	117.9	87.3	117.8	87.5

5. Effect of the coating method on the coating uniformity of the final materials

In order to compare the coating effect between the one-time sintering method and the conventional dry coating method, 3 wt.% ZrO_2-coated $LiMn_2O_4$ materials via the above two coating methods were synthesized, respectively. It can be seen from Fig. S4(d, f) that both of the particle surfaces are coated by many small ZrO_2 particles ranging from 30~40 nm, and the coating uniformity of the one-time sintering coating method is similar to that of the conventional coating method.

6. The morphologies of different amounts of SiO_2- and TiO_2-coated $LiMn_2O_4$

The sintering products of different amounts of SiO_2-coated Mn_3O_4&Li_2CO_3 and TiO_2-coated Mn_3O_4&Li_2CO_3 mixtures calcined at 550 ^oC and 800 ^oC were characterized using SEM. Fig. S6 shows the SEM images of the pristine $LiMn_2O_4$ and 1, 3, 5 wt.% SiO_2-coated $LiMn_2O_4$ materials calcined at 550 ^oC and 800 ^oC. When the sintering temperature is 550 ^oC, it is clearly seen that there are many coating materials on the surface of $LiMn_2O_4$ particles. Moreover, the coating integrity of 1 wt.% coating amount is inferior to that of 3 and 5 wt.% coating amount. When the sintering temperature increases to 800 ^oC, the coating layers completely disappear, no matter the coating amount is 1, 3, or 5 wt.%. It demonstrates that Si has diffused into the bulk crystals of $LiMn_2O_4$ at 800 ^oC. As illustrated in Fig. S7, the same results for different amounts of TiO_2-coated $LiMn_2O_4$ are also obtained.

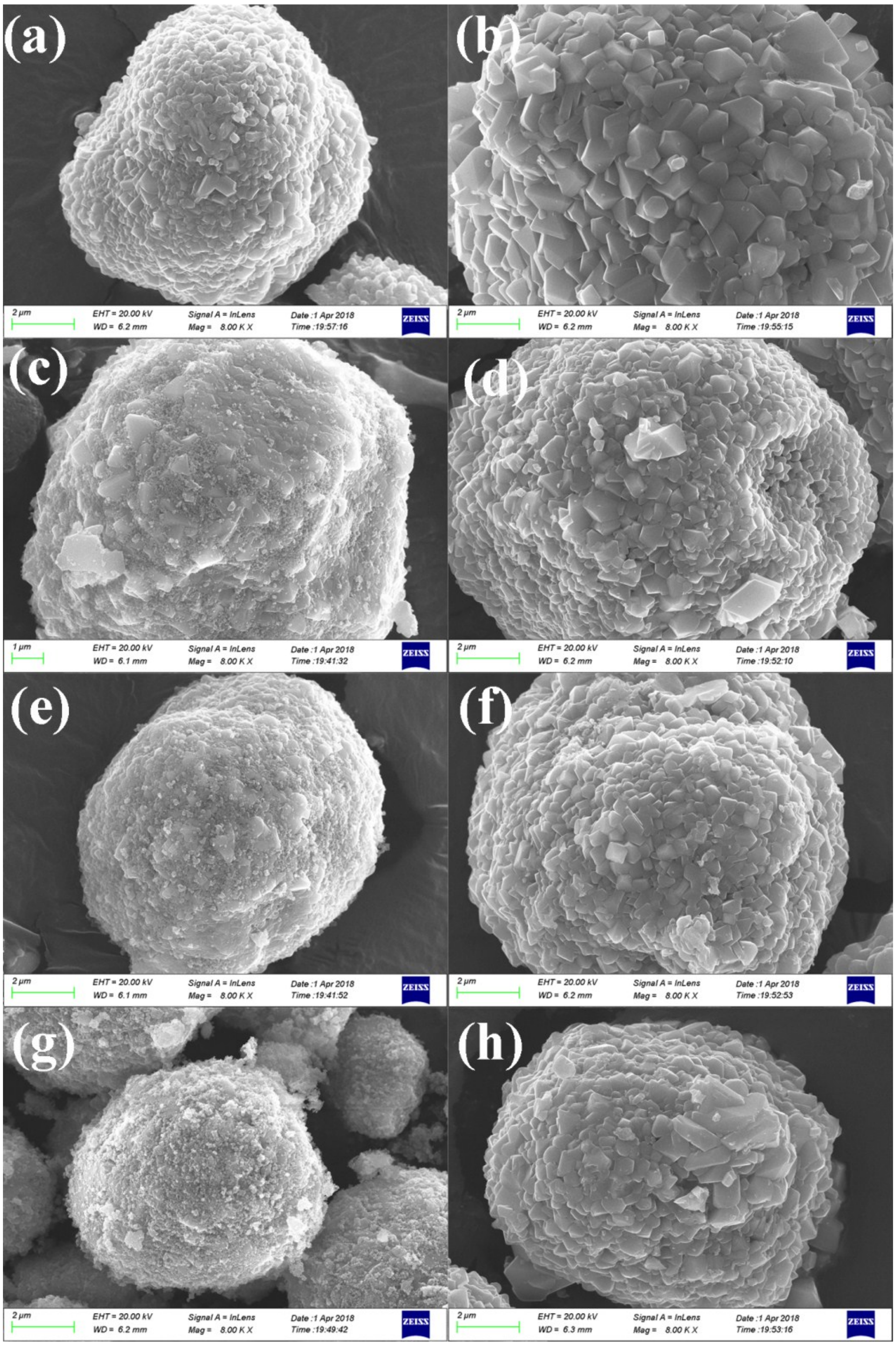

Fig. S6. SEM images of the sintering products of 0, 1.19, 3.56 and 5.93 wt.% SiO_2-coated Mn_3O_4@Li_2CO_3 mixtures calcined at different temperatures: (a, c, e, g) 550 ^oC; (b, d, f, h) 800 ^oC.

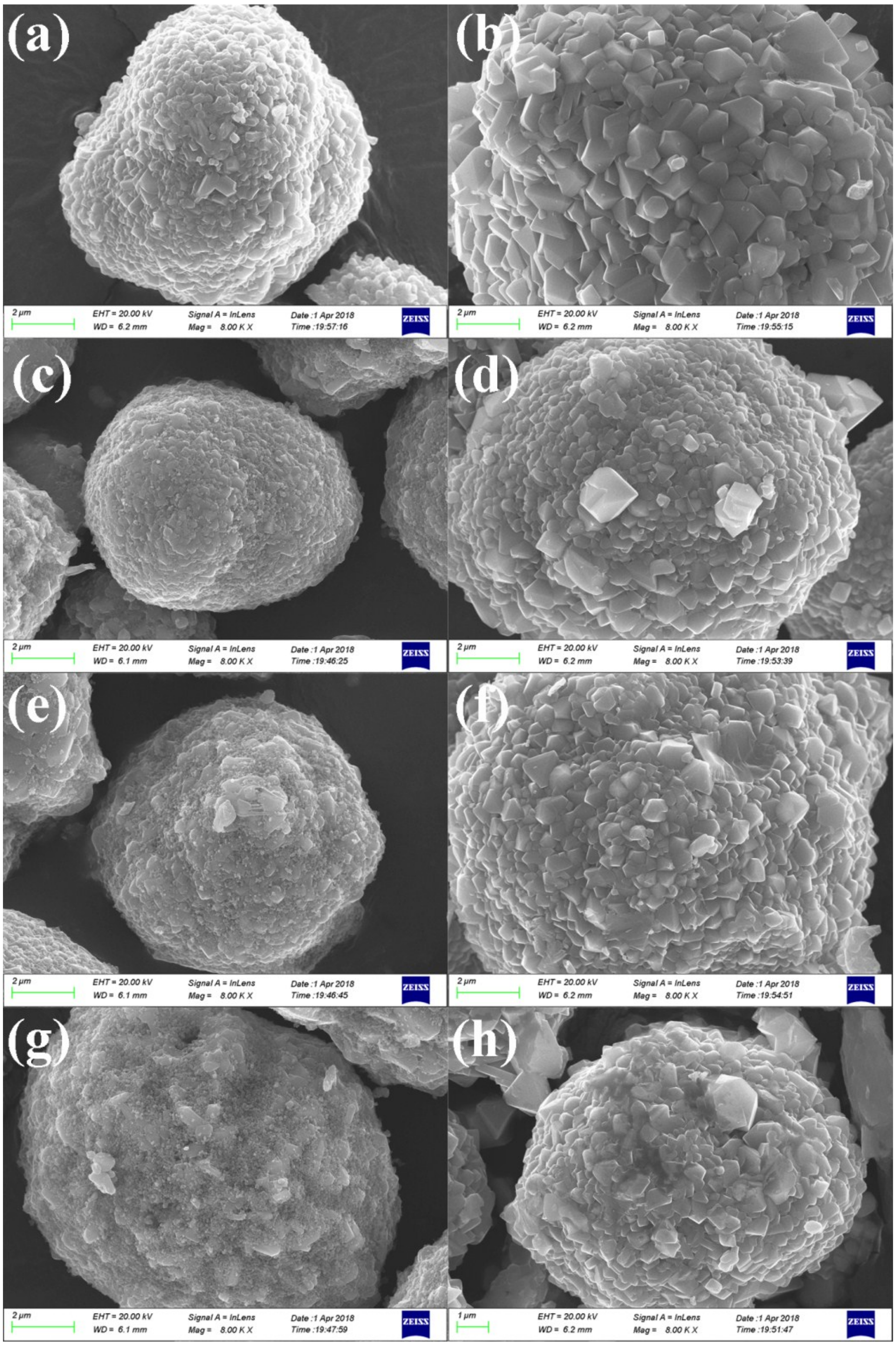

Fig. S7. SEM images of the sintering products of 0, 1.19, 3.56 and 5.93 wt.% TiO_2-coated Mn_3O_4@Li_2CO_3 mixtures calcined at different temperatures: (a, c, e, g) 550 ^oC; (b, d, f, h) 800 ^oC.

7. Crystal structures of 3 wt.% SiO_2-coated $LiMn_2O_4$ and 3 wt.% TiO_2-coated $LiMn_2O_4$ calcined at 550 °C

XRD was employed to measure the crystal structures of the sintering products of 3.56 wt.% SiO_2-coated Mn_3O_4&Li_2CO_3 and 3.56 wt.% TiO_2-coated Mn_3O_4&Li_2CO_3 heat-treated at 550 °C.

As illustrated in Fig. S8a, the major diffraction peaks of the samples indexed to the spinel $LiMn_2O_4$ are observed, but the diffraction peaks indexed to SiO_2 and Mn_2O_3 are also observed, indicating that the SiO_2 coating layer is still maintained on the surface of $LiMn_2O_4$ particles, which can be proven from the SEM image in Fig. S4e. On the other hand, there are still some Mn_3O_4 unreacting with Li_2CO_3 to form $LiMn_2O_4$. What needs to be explained is that the unreacting Mn_3O_4 would be transformed to Mn_2O_3 in O_2 at 550 °C.

The same XRD result is also obtained from 3 wt.% TiO_2-coated $LiMn_2O_4$ materials calcined at 550 °C. As illustrated in Fig. S8b, the diffraction peak indexed to TiO_2 can be observed, indicating that the TiO_2 coating layer is still maintained on the surface of $LiMn_2O_4$ materials, which is well agreement with the result obtained in Fig. S6e.

The above results demonstrate that the diffusing rates of Si and Ti ions into the crystal lattice of $LiMn_2O_4$ are very slow at 550 °C, so that the SiO_2 and TiO_2 coating layers can be maintained on the surface of $LiMn_2O_4$ particles.

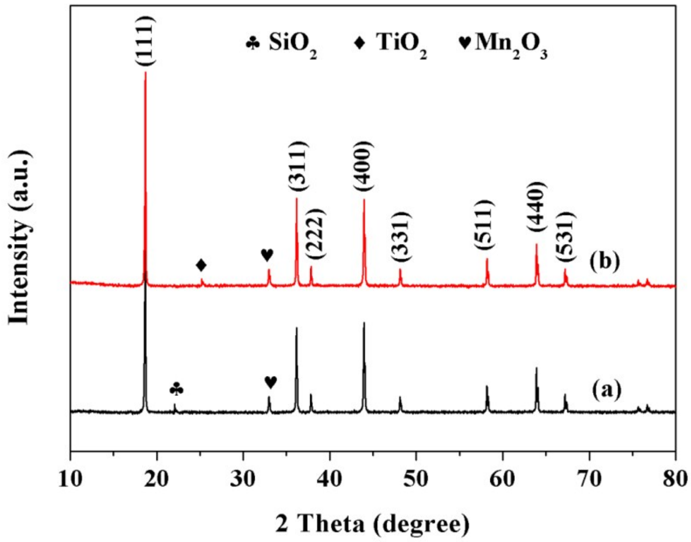

Fig. S8. XRD patterns of the sintering products of (a) 3.56 wt.% SiO_2-coated Mn_3O_4@Li_2CO_3 and (b) 3.56 wt.% TiO_2-coated Mn_3O_4@Li_2CO_3 calcined at 550 °C.

8. Crystal structures of different amounts of SiO_2- and TiO_2-coated $LiMn_2O_4$ calcined at 800 °C

XRD was employed to investigate the crystal structures of the sintering products of SiO_2-coated Mn_3O_4&Li_2CO_3 and TiO_2-coated Mn_3O_4&Li_2CO_3 heat-treated at 800 °C for 20 h.

As illustrated in Fig. S9a, all the diffraction peaks can be indexed to $LiMn_2O_4$ (JCPDF file No. 35-0782) with the spinel structure. Additionally, there is no diffraction peaks indexed to SiO_2 observed, indicating that Si has diffused into the bulk crystal to form $LiMn_{2-x}Si_xO_4$ phase. Fig. S9b shows the variation trend of lattice constant of the $LiMn_{2-x}Si_xO_4$ samples. In can be seen from Fig. S9b that the crystal lattice constant presents a trend of gradual increase with the increasing of Si-doping content. It is very interesting to find the unusual result since the ionic radius of Si^{4+} (r = 0.40 Å) is smaller than that of Mn^{4+} (r = 0.53 Å) in an octahedral site. According to the existing literatures (J. Power Sources, 2012, 216: 482-488; J. Mater. Chem. A, 2013, 1: 10857-10862.), the addition of Si^{4+} ions can increase the length of Mn-O bonding, angle of O-Mn-O and size of MnO_6 octahedra in the Si-doped $LiMn_2O_4$ samples, which leads to the more expanded and regular MnO_6 octahedra. Therefore, it is not difficult to understand the increase of the crystal lattice constant.

Fig. S9c shows the XRD patterns of the sintering products of 0, 1.19, 3.56 and 5.93 wt.% TiO_2-coated Mn_3O_4@Li_2CO_3 mixtures calcined at 800 °C for 20 h. All diffraction peaks can be indexed to $LiMn_2O_4$ (JCPDF file No. 35-0782) with the spinel structure without any impurity phase, which indicates that Ti has substituted partial Mn in the spinel structure. Simultaneously, as seen from Fig. S8d, the lattice constant increases gradually with Ti content increasing because of the expanded crystal cell of $LiMn_2O_4$, which is due to the replacement of Mn^{4+} (r = 0.53 Å) with a larger Ti^{4+} (r = 0.605 Å) on octahedral sites and the decrease in the manganese average oxidation state.

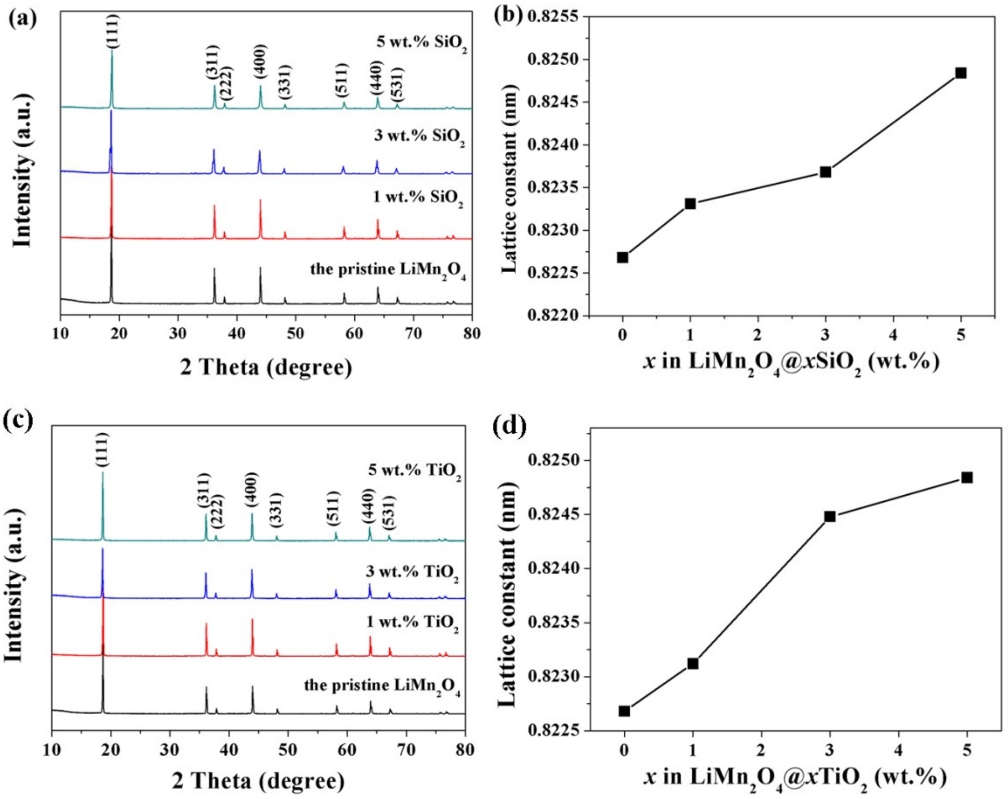

Fig. S9 (a) XRD patterns and (b) the lattice constant of the sintering products of 0, 1.19, 3.56 and 5.93 wt.% SiO_2-coated Mn_3O_4@Li_2CO_3 mixtures calcined at 800 ^oC for 20 h；(c) XRD patterns and (d) the lattice constant of the sintering products of 0, 1.19, 3.56 and 5.93 wt.% TiO_2-coated Mn_3O_4@Li_2CO_3 mixtures calcined at 800 ^oC for 20 h.

9. Effect of the sintering temperature and time on crystal structures of the sintering products

Influence of sintering temperature and time on the structure and performance of these materials was investigated in detail. Fig. S10 shows XRD patterns of the sintering products of different amounts of ZrO_2-coated Mn_3O_4&Li_2CO_3 calcined at different temperatures. When the sintering temperature is between 550 °C and 600 °C, the main diffraction peaks indexed to cubic spinel $LiMn_2O_4$ can be easily observed, indicating that Li_2CO_3 has reacted with Mn_3O_4 to form $LiMn_2O_4$. However, the intensity of diffraction peaks attributed to $LiMn_2O_4$ is low and the diffraction peaks indexed to Mn_2O_3 can be observed, indicating that Li_2CO_3 has not completely reacted with Mn_3O_4. Some unreacted Mn_3O_4 are transformed into Mn_2O_3. When the sintering temperature is between 650 °C and 700 °C, the cubic spinel structure has been formed, but the intensity of diffraction peaks is low and half-peak width is big, which demonstrates that the spinel crystals are not well-ordered. When the sintering temperature is raised to 750~850 °C, the intensity of diffraction peaks becomes stronger with the temperature increasing. As to 3 wt.% and 5 wt.% ZrO_2-coated $LiMn_2O_4$ materials, when the sintering temperature is between 550 °C and 850 °C, the diffraction peaks of ZrO_2 can always be observed. Therefore, three conclusions can be obtained: 1) ZrO_2 has not reacted with Li_2CO_3 to form Li_2ZrO_3; 2) Most of Zr ions have not diffused into the bulk crystal of $LiMn_2O_4$, so the ZrO_2 coating layer can still be maintained even after long-term high-temperature heat-treatment; 3) As the temperature increases, the intensity of diffraction peaks indexed to ZrO_2 becomes stronger, which probably shows that the ZrO_2 coating layer becomes denser after high-temperature heat-treatment.

Fig. S11 shows the XRD patterns of 3 wt.% ZrO_2-coated $LiMn_2O_4$ calcined at 800 °C for 20 h and 40 h, respectively. It can be seen from Fig. S10 that the main diffraction peaks of two samples can be indexed to cubic spinel $LiMn_2O_4$ structure, *Fd*3*m* space group. Additionally, as the sintering time increases, the location of diffraction peaks of $LiMn_2O_4$ changes little and the diffraction peaks attributed to ZrO_2 can still be easily observed, indicating that the ZrO_2 coating layer can still be maintained even after calcination at 800 °C for 40 h.

From XRD data, we have obtained that most of ZrO_2 have not diffused into the inner part of $LiMn_2O_4$ particles even after long-term high-temperature calcination. However, although the melting point of ZrO_2 is high to 2700 °C, there are still some Zr ions diffusing into the

crystal lattice to form $LiMn_{2-x}Zr_xO_4$ phase. This is because solid-state diffusion reaction still can take place even if the sintering temperature can't reach the melting point of the raw materials.

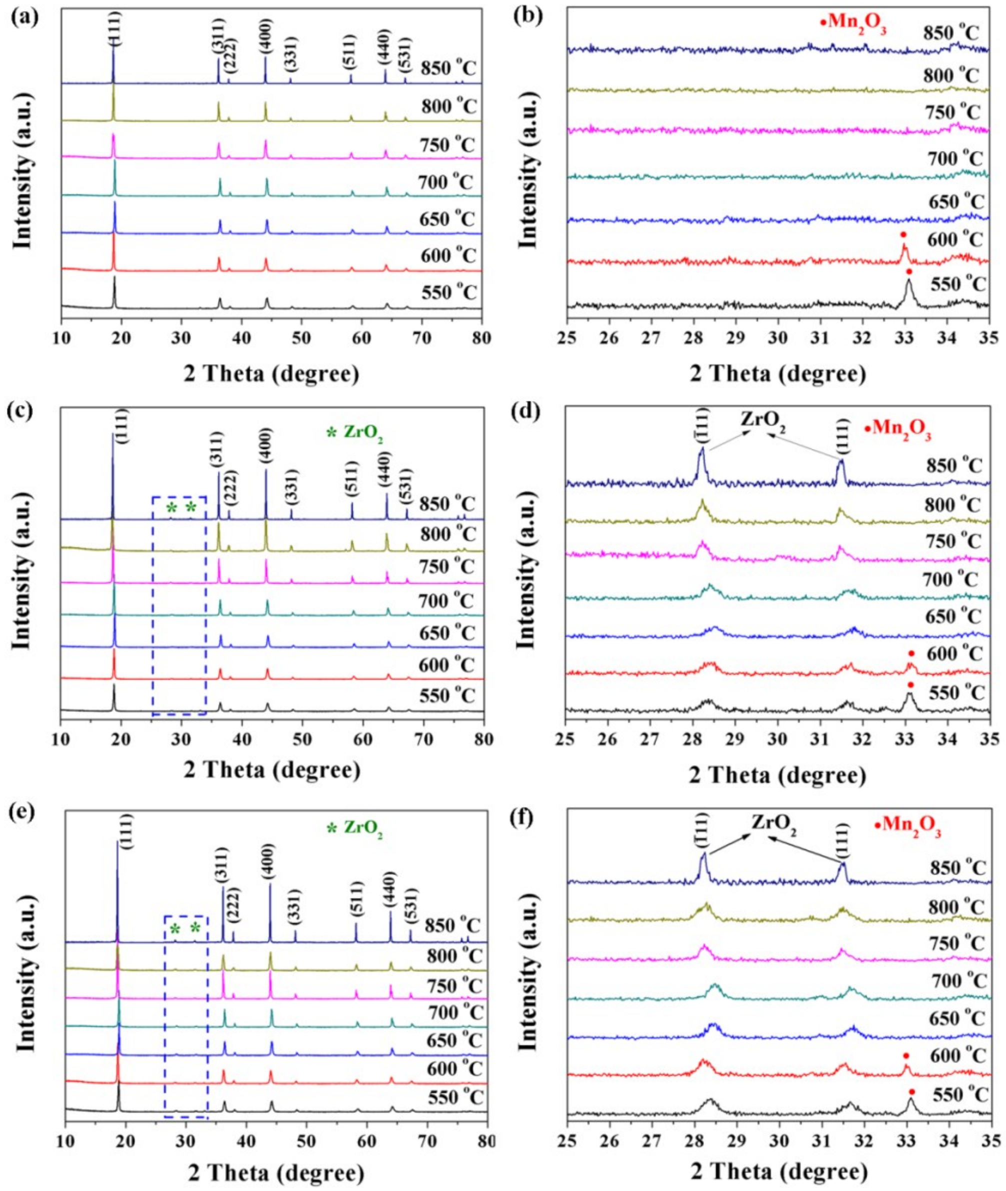

Fig. S10. XRD patterns of the sintering products of (a, b) Mn_3O_4&Li_2CO_3, (c, d) 3.56 wt.% ZrO_2-coated Mn_3O_4&Li_2CO_3, and (e, f) 5.93 wt.% ZrO_2-coated Mn_3O_4&Li_2CO_3 calcined at different temperatures.

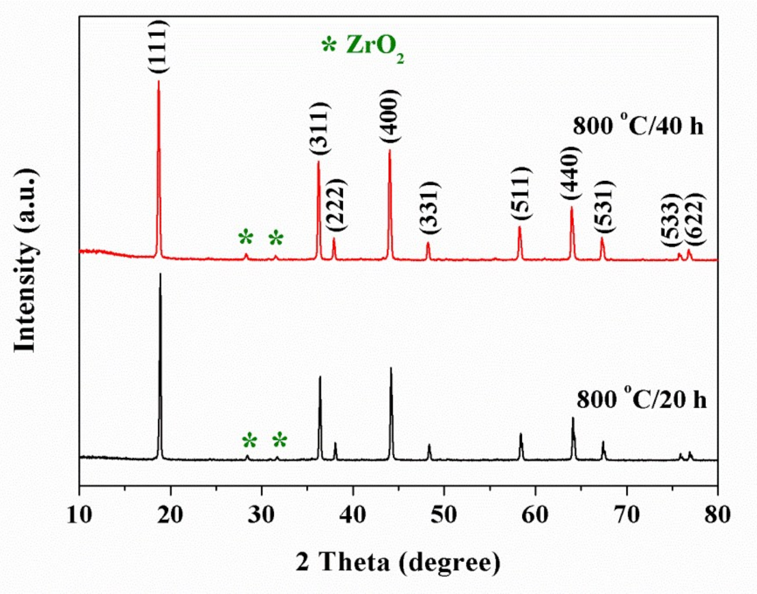

Fig. S11. XRD patterns of 3 wt.% ZrO_2-coated $LiMn_2O_4$ calcined at 800 °C for 20 h and 40 h, respectively.

Fig. S12 shows the cyclic performance of the pristine $LiMn_2O_4$ materials calcined at different temperatures for 20 in O_2 atmosphere. The charge/discharge rate is 0.2 C (24 mA·g^{-1}) for the first 4 cycles and 1 C (120 mA·g^{-1}) for the following cycles. In the voltage range of 3.0~4.3 V (*vs*. Li^+/Li), the initial specific discharge capacities of the $LiMn_2O_4$ samples calcined at 750 °C, 800 °C, and 850 °C at 0.2 C are 119.8, 121.3, and 119.4 mAh·g^{-1}, respectively. The 300th capacity retentions of $LiMn_2O_4$ samples calcined at 750 °C and 800 °C are 81.4 and 88.0%, respectively. For the sample calcined at 850 °C, the discharge capacity drops rapidly in the charge-discharge processes with the 100th capacity retention of only 60.7%. From the above results, we can draw a conclusion that the optimal synthesizing temperature of $LiMn_2O_4$ is 800 °C.

Fig. S13 shows the cyclic performance of $LiMn_2O_4$ samples calcined at 800 °C for 20 h and 40 h. The initial specific discharge capacities of the samples calcined for 20 h and 40 h are 121.3 and 122.5 mAh·g^{-1} with the 300th capacity retention of 88.0% and 86.6%, respectively. The above result shows that the discharge capacity slightly increases and the capacity retention slightly decreases with the sintering time increasing. The reason is probably that more Li^+ ions evaporate in the long-term high-temperature heat-treatment, resulting in the lower Li/Mn ratio, thus the discharge capacity slightly increases and the capacity retention slightly decreases.

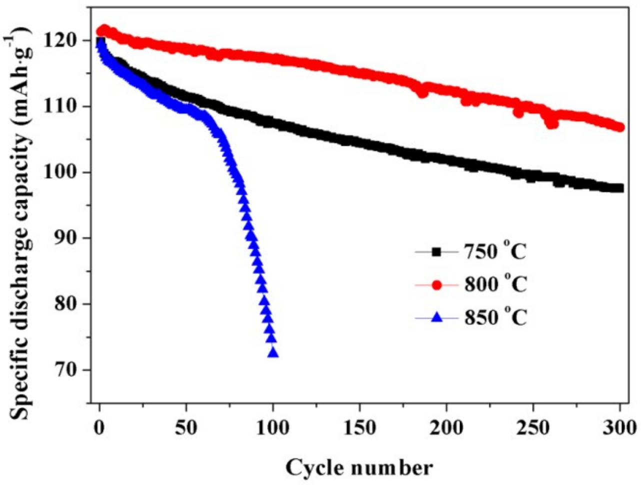

Fig. S12. Cyclic performance of $LiMn_2O_4$ calcined at different temperatures for 20 h in O_2 atmosphere.

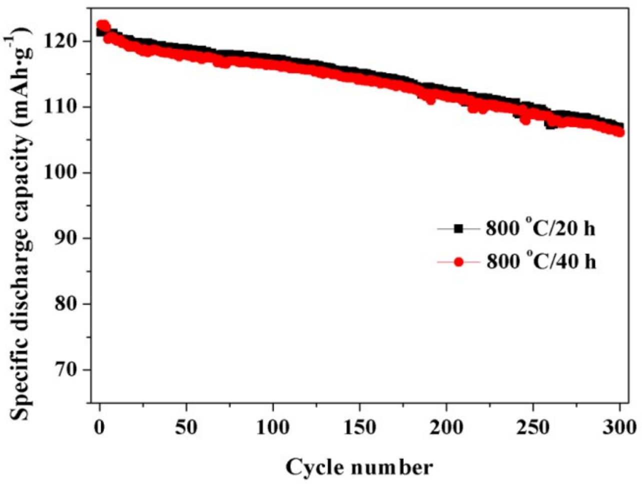

Fig. S13. Cyclic performance of $LiMn_2O_4$ calcined at 800 °C for 20 h and 40 h in O_2 atmosphere.

10. SEM and XRD characterizations of 3 wt.% ZrO_2-coated $LiMn_2O_4$ electrode post electrochemical testing

3 wt.% ZrO_2-coated $LiMn_2O_4$ electrode cycled for 150 cycles at 55 °C was characterized using SEM and XRD. It is obviously seen from Fig. S14 that there are many small particles with the particle size of 30~40 nm on the surface of $LiMn_2O_4$ particle, which demonstrates that the coating layer still exists after electrochemical testing.

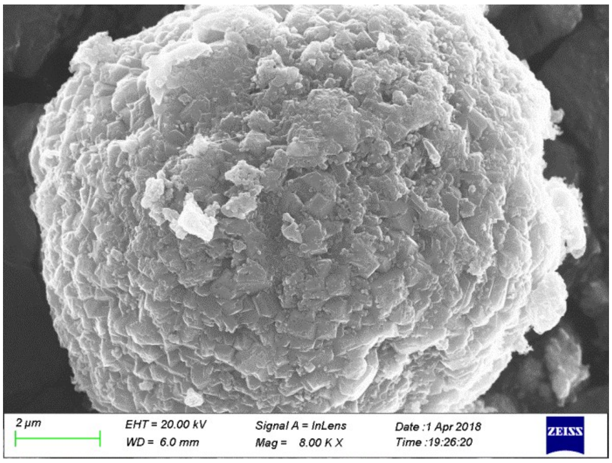

Fig. S14. SEM image of 3 wt.% ZrO_2-coated $LiMn_2O_4$ after 150 cycles at 55 °C.

Fig. S15 shows the XRD patterns of 3 wt.% ZrO_2 coated $LiMn_2O_4$ electrode after 150 cycles at 55 °C. The major diffraction peaks are in good agreement with that obtained from JCPDF file No. 35-0782, indicating that the cubic spinel structure of $LiMn_2O_4$ material is still maintained post electrochemical test. Additionally, the diffraction peaks located at 28.2 ° and 31.5 ° indexed to ZrO_2 can still be observed, which implies that the ZrO_2 coating layer does not disappear.

Based on the analysis of SEM and XRD data, we can conclude that the ZrO_2 coating layer on the surface of $LiMn_2O_4$ particles is very stable to the volume changes brought by the long-term charge-discharge processes.

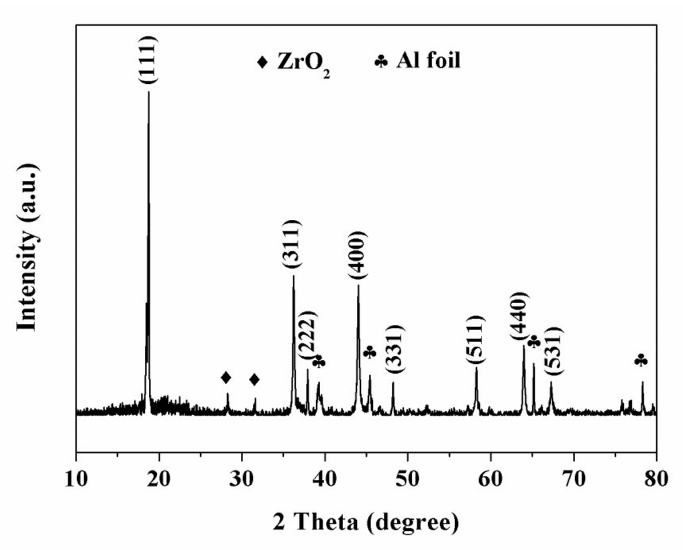

Fig. S15. XRD pattern of 3 wt.% ZrO_2-coated $LiMn_2O_4$ electrode after 150 cycles at 55 ^oC.
